# Cytotoxicity Studies of Novel Combretastatin and Pterostilbene Derivatives

**DOI:** 10.1155/2014/320895

**Published:** 2014-08-03

**Authors:** Joanna Jakubowska, Justyna Mikuła-Pietrasik, Krzysztof Książek, Hanna Krawczyk

**Affiliations:** ^1^Department of Organic Chemistry, Faculty of Chemistry, Warsaw University of Technology, Noakowskiego 3,00-664 Warsaw, Poland; ^2^Department of Pathophysiology, Laboratory of Gerontology, Poznań University of Medical Sciences, ul. Święcickiego 6, 60-781 Poznań, Poland

## Abstract

We synthesised seven 2-aminestilbenes with methoxy substitents in reactions of dinitrostilbenes with sodium azide. In order to study the positioning of the nitro groups, the optimum structure of obtained stilbenes using the DFT B3LYP/6-311++G(2d,p) method was calculated. Very interesting aspect of this regioselectivity reaction is the fact that in all substrates and synthetized compounds the nitro groups in position 2 were not coplanar whereas the* para*-nitro groups were coplanar with respect to the benzene ring. Due to unique features of stilbene derivatives, such as antitumor agents, we undertook the studies on the biological properties of new stilbene derivatives. Using five cancer cell lines, we investigated the effects of 2-aminestilbenes with methoxy substitents on cell growth.

## 1. **Introduction**

**Figure 1:**
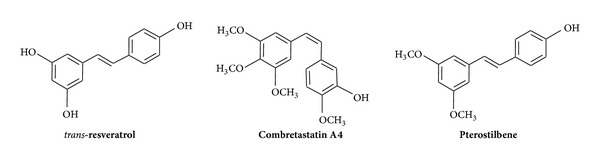
The structure of* trans*-resveratrol, of combretastatin A4, and of pterostilbene.

“The drug market is still dominated by small molecules and more than 80% of the clinical development of drug candidates in the top 20 pharmaceutical firms is still based on small molecules. The high cost of developing and manufacturing “biological drugs” will contribute to leaving an open space for drugs based on cheap small molecules” [1]. The stilbenoids, which are actually small molecules, possess a wide spectrum of properties which allow considering these compounds as potentially important drugs with a broad therapeutic window.* Trans*-resveratrol ((*E*)-3,5,4′-trihydroxystilbene) (Figure 1) may be considered to be used as a cancer chemopreventive agent showing antioxidant activity and causing upregulation of NO production [2, 3].* Trans*-resveratrol therapy may prevent the hypertensive response and the relaxation response to acetylcholine [4]. The* trans*-resveratrol also exhibits cardioprotective effects and anticancer properties, as suggested by its ability to suppress the proliferation of a wide variety of tumour cells, including lymphoid and myeloid cancers, multiple myeloma, cancers of the breast, prostate, stomach, colon, pancreas, and thyroid melanoma, head and neck squamous cell carcinoma, ovarian carcinoma, and cervical carcinoma [5].

Combretastatin A4 (a class of natural phenols) is potential new vascular disrupting agent (VDAs) and vascular targeting agent (VTAs) [6] and shows also a remarkable ability to inhibit gastric tumour metastasis and enhanced antitumor immune reactivity [7]. This compound may provide an effective means of treatment for refractory organ-infiltrating leukemias and is potentially important for optimizing the therapeutic combination of vascular targeting agents with radiotherapy. Pterostilbene (3,5-dimethoxy-4′-hydroxy-(*E*)-stilbene) possesses lipid and glucose lowering properties useful in the treatment of resistant haematology malignancies, exhibits antioxidant capacity, and demonstrates concentration-dependent anticancer activity [8–12]. It is also notable that stilbenes with nitro groups [13] or amine group [14] exhibit anticancer properties on HL-60 leukaemia cell line.

Due to the widespread use and importance of stilbenes, which are small molecules, new and active stilbenes are still being looked for. In continuation of our studies on stilbenes [15] we have synthesised novel group of stilbenes with methoxy groups in one ring and amine and nitro group in the second ring (**1b**–**7b**). So far, no one has studied the biological activity of these stilbenes. Therefore, we investigated cytotoxicity of these compounds. 

## 2. **Material and Methods**

### 2.1. General Procedure for the Reductive Amination of Dinitrostilbenes with NaN_3_

**3(a)** (1.45 mmol) and NaN_3_ (164 mg, 2.52 mmol) in DMF (15 mL) were sequentially added to a three-necked flask (25 mL) fitted with a condenser (see Supplementary Material available online at http://dx.doi.org/10.1155/2014/320895). The mixture was stirred at 120°C for 20 h and concentrated in vacuo. The residue was purified by flash column chromatography on silica gel (toluene → toluene: MeOH, 4 : 1).

### 2.2. Cancer Cell Lines

Colorectal cancer cells SW480, breast cancer cells MDA-MB-231, and lung cancer cells A549 were purchased from the American Type Culture Collection (Rockville, MD) while ovarian cancer cells SKOV-3 and pancreatic cancer cells PSN-1 were obtained from the European Collection of Cell Cultures (Porton Down, UK). All cancer cell lines were propagated in RPMI-1640 medium (Sigma-Aldrich Corp., St. Louis, MO, USA) supplemented with 10% FBS, L-glutamine (2 mM), penicillin (100 U/mL), and streptomycin (100 *μ*g/mL).

The half maximal inhibitory concentrations (IC_50_) for** 1**–**7** stilbenes were calculated in GraphPad Prism 5 programme.

### 2.3. Measurement of Cell Growth Using MTT Assay

Cancer cells were seeded into 96-well plates at a density of 5 × 10^4^ cells/cm^2^ and maintained in standard growth medium until reaching confluency. Then the cells were incubated with standard growth medium enriched in the appropriate stilbenes for 24 hours at 37°C. Afterwards the medium was removed and cells were carefully washed and incubated with a fresh medium containing 1.25 mg/mL of the MTT salt [3-(4,5-dimethylthiazol-2-yl)-2,5-diphenyltetrazolium bromide] for 24 hours at 37°C. The formazan product generated was solubilized by the addition of 20% sodium dodecyl sulphate and 50%* N,N*-dimethylformamide. Absorbance of the converted dye was recorded at 595 nm with a reference wavelength of 690 nm.

## 3. **Results and Discussion**

### 3.1. Chemical Synthesis

In our last work we found that the reaction between a derivative of 2,4-dinitrostilbene and sodium azide always gave the corresponding 2-amino-4-nitrostilbene as the sole product instead of the expected azidonitrostilbene [15]. We also optimized the following conditions for this unexpected transformation: (1) sodium azide was required, as without this reagent no product was observed, (2) 120°C and DMF or DMSO were the best combination of temperature and solvent, and (3) the reaction proceeded in air or under an argon atmosphere. In order to see the effect of various methoxy groups in the first ring and amine and nitro groups in the second ring on the biological properties, we have subjected stilbene reaction described by us [15] to obtain easily and quickly novel derivatives of methoxystilbene.

**Scheme 1:**
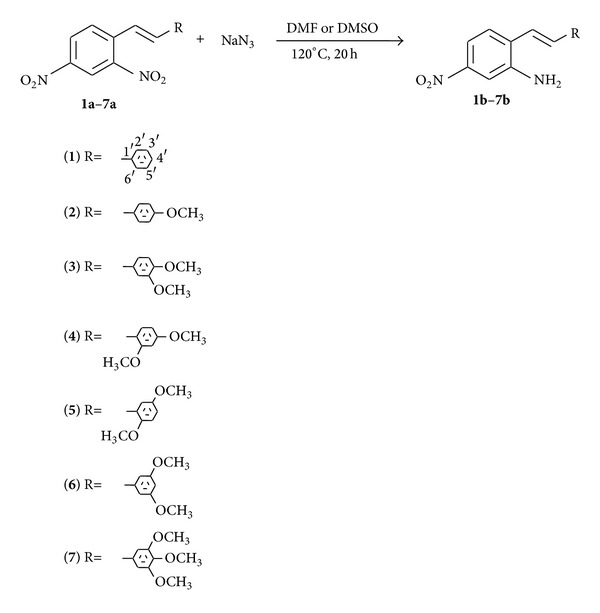
The formation of aminostilbenes** 1b**–**7b** from nitrostilbenes** 1a**–**7a** under azidation conditions.

Our general approach to synthesis of aminostilbenes was to perform a reaction of dinitro stilbenes** 1a**–**7a** with sodium azide to obtain regioselective 2-amine-4-nitrostilbenes** 1b**–**7b** (Scheme 1). All the synthesized stilbenes were obtained with a good yield, 75%–82%, and this method could be used as a preparative method for selective formation of 2-amino-4-nitrostilbenes. A very interesting aspect of this regioselectivity reaction [15] is the fact that in all the synthesized compounds the nitro groups in position 2 were not coplanar (Table 1). In order to study the positioning of the nitro groups, the optimum structure of** 1a**–**7a** using the DFT B3LYP/6-311++G(2d,p) method was calculated (see Supplementary Material). As in the case of the stilbenes obtained previously, [15] the* ortho*-nitro group in position 2 of** 1a**–**7a** was rotated around the C–N axis by 30.6°–37.4° (Table 1), whereas the* para*-nitro group was coplanar with respect to the benzene ring. The same situation is for obtained stilbene** 1b**–**7b** ( rotated around the C–N axis by 14.0°–35.4° and the* para*-nitro group was coplanar with respect to the benzene ring). From the analysis of DFT calculation (see Supplementary Material) we could conclude that stilbenes** 1b**–**7b**, that were used in investigation into cytotoxicity, had approximately the same scaffold.

### 3.2. Cytotoxicity

The group of synthesised stilbenes was compounds with methoxy groups in one ring and amine and nitro groups in the second ring. It is known that the shared structural feature present in combretastatin analogues is a 3,4,5-trimethoxyaryl ring (A-ring), widely recognized to be essential for biological activity. However, it has been recently reported that this is not necessarily the case [16–22]. Beale et al. [22] modified the A-ring of 3,4,5-trimethoxyaryl ring and discovered that 3,5-dibromo- and 3,5-diiodo-substitution of the A ring still took place and even improved upon the activity of one of these compounds, in human umbilical vein endothelial cells (HUVECs) and ovarian cancer cells (SK-OV-3 and paclitaxel-resistant SK-OV-3TR). Also the stilbenes with nitro group [13] or amine group [14] show anticancer effect on HL-60 leukemia cell line.

Taking into account these leads and the analysis of the influence of substituent on the ring we have undertaken investigation into cytotoxicity of** 1b**–**7b** synthesized stilbenes. These compounds had methoxy substituents in both different amounts and positions in A-ring, whereas amine and nitro groups were always in the same positions in ring B, that is, in positions 2 and 4, respectively (Scheme 1). Stilbene** 1b** was without substituents in ring A and was a referenced scaffold. Using five cancer cell lines, we investigated the effects of** 1b**–**7b** cell growth on the cell cycle. The results of cell proliferation were obtained using three replicate experiments of MTT assay. The five graphs presented the percentage of cell growth in correlation with the concentration. In the next five diagrams the following relationship was shown: % cell growth of stilbene** 1b** (referenced scaffold) minus % cell growth of stilbene** 2b**–**7b**, respectively, and concentration. We performed this operation assuming that all the tests were carried out for the same concentration, at the same time, under the same conditions, and with the same referenced scaffold (Table 1). In this way, we analysed the effect of stilbene substituents occurring successively in the skeleton.

#### 3.2.1. Breast Cancer Cells MDA-MB-231

**Figure 2:**
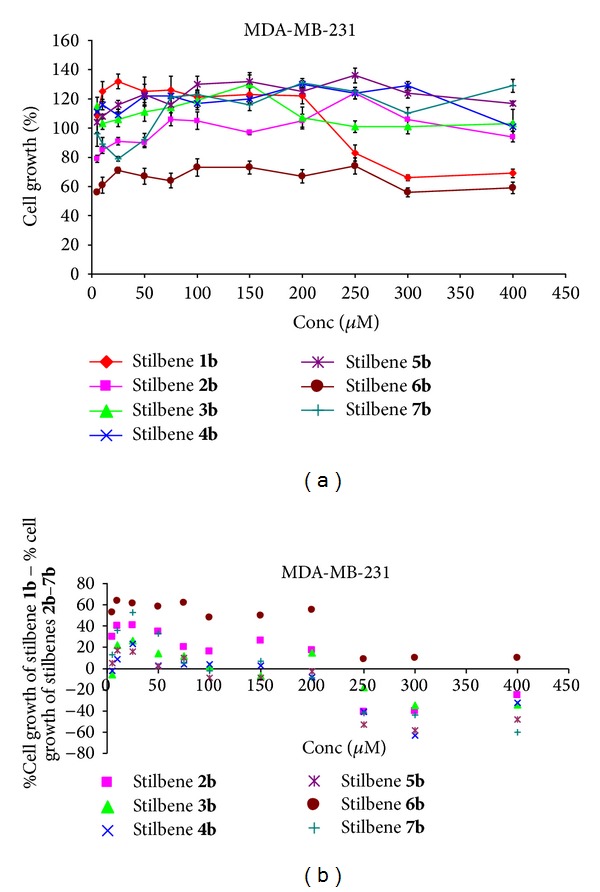
(a) Dose response curves for stilbenes** 1b**–**7b** in breast cancer cells MDA-MB-23. Data are expressed as percentage growth of vehicle treated controls. Values represent the means ± SEM for three separate experiments carried out in triplicate; (b) the relationship: % cell growth of stilbene** 1b** (referenced scaffold) minus % cell growth of stilbene** 2b**–**7b,** respectively, and concentration.

Stilbene** 1b** in concentrations of 5–200 *μ*M induced tumour cell proliferation and above these concentrations it was cytotoxic (Figure 2(a)). In the case of stilbene** 2b**, an introduction of the one methoxy group of the ring A in the 4-position caused a cytotoxic effect at low concentrations (10 *μ*M) and for the other concentrations (except 250 *μ*M—proliferation) it had no effect (Figure 2(a)).

Modification of the ring A of the substituent in the concentration range of 5–200 *μ*M caused cytotoxic effect (in comparison with the stilbene ring). Above this concentration we observed the effect of proliferation (Figure 2(b)). Regarding stilben** 3b**, an introduction of two methoxy groups to the ring A (at positions 3′, 4′) is not recommended—for the whole range of concentrations the cell proliferation occurred (Figure 2(a)). In the 10–75 *μ*M the cytotoxicity increased, compared to nonsubstituted stilbene (Figure 2(b)). In the case of stilbene** 4b**, introduction of the two methoxy groups to the ring A (at positions 2′, 4′) was undesired for the whole range, because of the cell proliferation (Figure 2(a)). For a concentration of 25 *μ*M there occurred a slight increase in cytotoxicity compared to nonsubstituted stilbene (Figure 2(b)). Introduction of the methoxy groups to the ring A (at positions 2′, 5′) in stilbene** 5b** is not recommended, because the cell proliferation occurred in the whole range of concentrations (Figure 2(a)). In the range of 5–25 *μ*M there was a slight increase in cytotoxicity compared to nonsubstituted stilbene (Figure 2(b)). In the case of stilbene** 6b** an introduction of two methoxy groups to the ring A (at positions 3′, 5′) is recommended for the whole range of concentrations because of the increase in cytotoxicity, the largest for the concentrations of 5 *μ*M and 400 *μ*M (Figure 2(a)). In the case of this stilbene, the highest cytotoxic effect for 5–200 *μ*M was observed in relation to the unsubstituted stilbene (Figure 2(b)). Below this concentration there was a slight increase in toxicity. The presence of three methoxy groups in ring A (at positions 3′, 4′, 5′) in stilbene** 7b** had little effect on the cytotoxicity in the range of 5–50 *μ*M (Figure 2(a)). However, for higher concentrations the effect of proliferation could be observed. In relation to nonsubstituted stilbene there was a larger increase in the cytotoxic effect at concentrations of 10-50 *μ*M (Figure 2(b)).

#### 3.2.2. Pancreatic Cancer Cells PSN-1

**Figure 3:**
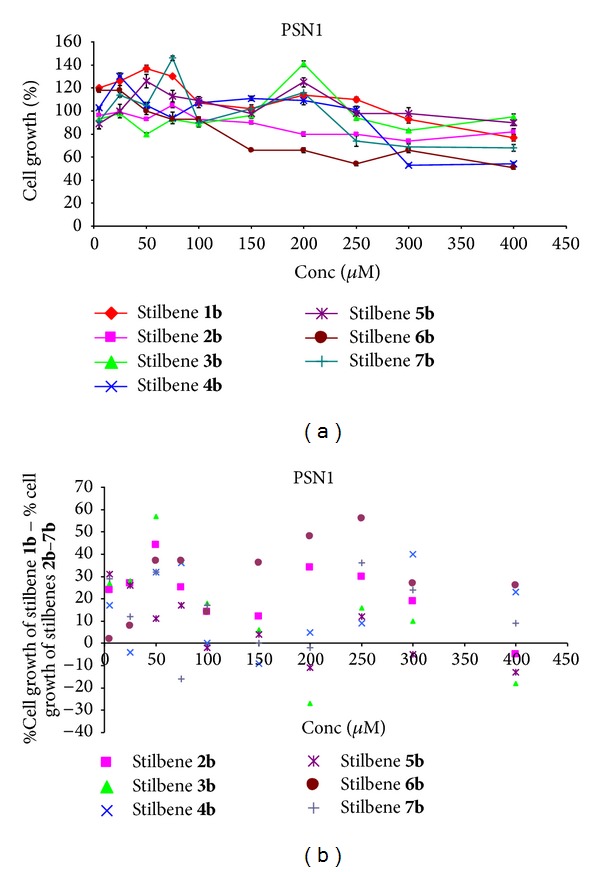
(a) Dose response curves for stilbenes** 1b**–**7b** in pancreatic cancer cells PSN-1. Data are expressed as percentage growth of vehicle treated controls. Values represent the means ± SEM for three separate experiments carried out in triplicate; (b) the relationship: % cell growth of stilbene** 1b** (referenced scaffold) minus % cell growth of stilbene** 2b**–**7b,** respectively, and concentration.

Stilbene** 1b** exhibited the proliferation effect for concentrations up to 300 *μ*M and the cytotoxic effect for higher concentrations (Figure 3(a)). In the case of stilbene** 2b** an introduction of the methoxy group to the ring A in the 4-position no cytotoxic effect was observed at concentrations greater than 100 *μ*M. For the remaining concentrations the cytotoxic effect was insignificant (Figure 3(a)). Introduction of a methoxy substituent at concentrations 5–300 *μ*M resulted in a significant cytotoxic effect (in comparison with the ring stilbene) (Figure 3(b)). Above this concentration the proliferation effect was observed. Introduction of two methoxy groups to the ring A (at positions 3′,4′) in stilbene** 3b** caused a slight cytotoxic effect (except for the concentration of 200 *μ*M) (Figure 3(a)). Regarding the impact of double methoxy group, except for the concentrations of 200 *μ*M and 400 *μ*M, an increase in the cytotoxic effect could be observed and for the concentration of 50 *μ*M, the highest in this series (Figure 3(b)). While considering stilbene** 4b**, an introduction of the methoxy groups to the ring A (at positions 2′, 4′) resulted in the proliferation of cells in the concentration range of 5–250 *μ*M and, for higher concentrations, in a strong cytotoxic effect (Figure 3(a)). The presence of the methoxyl substituents at concentrations of 25 and 150 *μ*M induced a proliferative effect (Figure 3(b)). For other concentrations the cytotoxic effect in comparison with unsubstituted stilbene could be observed. Two methoxy groups in the ring A (at positions 2′, 5′) were not suitable and in almost the whole range of concentrations the cell proliferation occurred (Figure 3(a)). For concentrations of 100, 200, and 400 *μ*M the proliferative effect could be observed, compared to nonsubstituted stilbene (Figure 3(b)). For the remaining concentrations a slight increase in the cytotoxic effect occurred. In the case of stilbene** 6b**, an introduction of two methoxy groups to the ring A (at positions 3′, 5′) increased the cytotoxic effect in ranges up to 100 *μ*M. Above these, a significant cytotoxic effect could be observed (Figure 3(a)). Ranging from 50 to 250 *μ*M, the high cytotoxic effect was noticed (in comparison with unsubstituted stilbene, Figure 3(b)). For stilbene** 7b**, introduction of three methoxy groups to ring A (at positions 3′, 4′, 5′) that had an impact on cell proliferation in the range of up to 200 *μ*M and above a cytotoxic effect could be observed (Figure 3(a)). For concentrations of 75, 150, and 200 *μ*M the proliferative effect was present in comparison with unsubstituted stilbene (Figure 3(b)). For increasing concentrations the cytotoxic effect was observed.

#### 3.2.3. Colorectal Cancer Cells SW480

**Figure 4:**
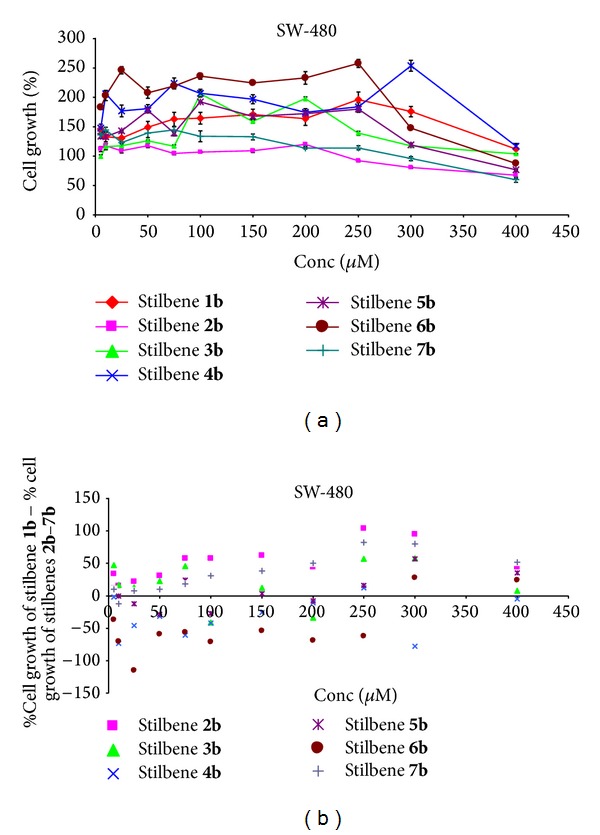
(a) Dose response curves for stilbenes** 1b**–**7b** in colorectal cancer cells SW480. Data are expressed as percentage growth of vehicle treated controls. Values represent the means ± SEM for three separate experiments carried out in triplicate; (b) the relationship: % cell growth of stilbene** 1b** (referenced scaffold) minus % cell growth of stilbene** 2b**–**7b,** respectively, and concentration.

For all the investigated stilbenes the cytotoxic effect was small, and only for stilbenes** 2b**,** 4b**,** 6b,** and** 7b** in a concentration of 400 *μ*M cytotoxic effect could be observed (Figures 4(a) and 4(b)).

#### 3.2.4. Lung Cancer Cells A549

**Figure 5:**
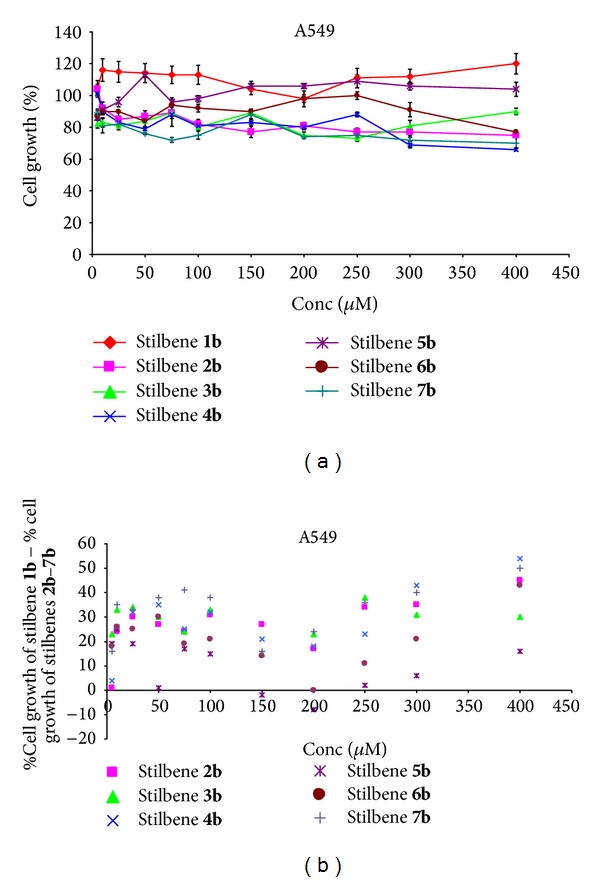
(a) Dose response curves for stilbenes** 1b**–**7b** in lung cancer cells A549. Data are expressed as percentage growth of vehicle treated controls. Values represent the means ± SEM for three separate experiments carried out in triplicate; (b) the relationship: % cell growth of stilbene** 1b** (referenced scaffold) minus % cell growth of stilbene** 2b**–**7b,** respectively, and concentration.

In the case of this cell line stilbene** 1b** caused cell proliferation (Figure 5(a)). Other stilbenes** 2b** (concentration above 25 *μ*M),** 3b** (full range),** 4b** (concentration above 25 *μ*M), and** 7b** (full range) induced a cytotoxic effect (Figure 5(a)). Influence of substituents (in comparison with the unsubstituted stilbene) for almost all stilbenes was associated with improving the cytostatic effect (except for stilbene** 5b** in concentration of 50 *μ*M, 150 *μ*M, and 200 *μ*M and stilbene** 6b** in concentration of 200 *μ*M (Figure 5(b)).

#### 3.2.5. Ovarian Cancer Cells SKOV-3

**Figure 6:**
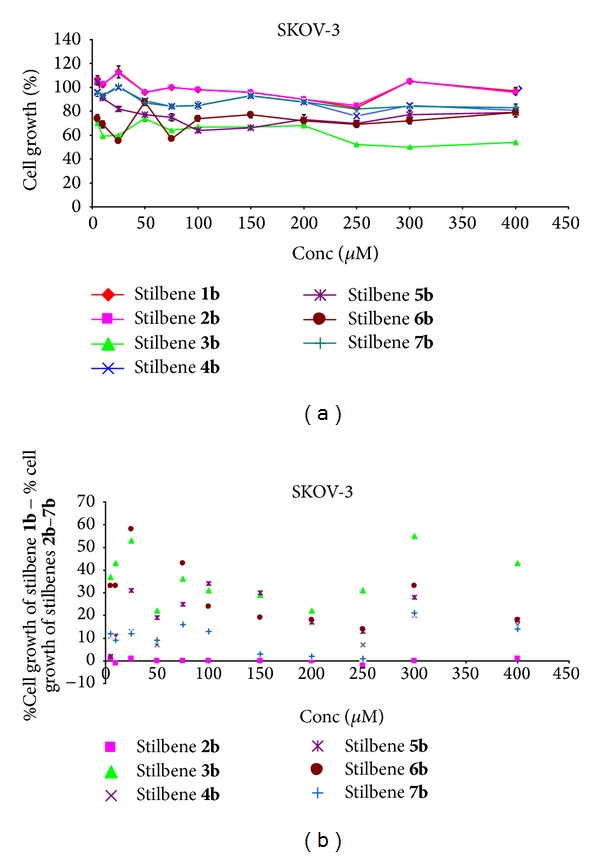
(a) Dose response curves for stilbenes** 1b**–**7b** in ovarian cancer cells SKOV-3. Data are expressed as percentage growth of vehicle treated controls. Values represent the means ± SEM for three separate experiments carried out in triplicate; (b) the relationship: % cell growth of stilbene** 1b** (referenced scaffold) minus % cell growth of stilbene** 2b**–**7b,** respectively, and concentration.

In this case, unsubstituted stilbene and stilbene substituted by one methoxy group exhibited a similar effect of being slightly cytotoxic in almost all the range of concentrations (Figures 6(a) and 6(b)). Other stilbenes in the whole range of concentrations exhibited cytotoxicity. The strongest effect was for stilbene** 3b** (Figure 6(a)). Accordingly, for stilbenes** 3b**–**7b** beneficial effects of substitution in all positions of the methoxy groups were observed. The largest effect occurred for stilbene** 6b** at the concentration of 25 *μ*M and for stilbene** 3b** at the concentration of 300 *μ*M (Figure 6(b)).

Using cancer cell lines described in the previous chapters we calculated the half maximal inhibitory concentrations (IC_50_) for** 1b**–**7b**. Any assays of viability were performed for five human carcinoma cell lines using three replicate experiments with errors shown as 95% confidence intervals (CI) (Table 2). In summary, investigated stilbenes had different activity (IC_50_ = 24.9–260 *μ*M/L) on all cell lines. For stilbenes** 1b**,** 3b** and** 4b** the growth inhibition effects were nonconverged. Stilbene** 2b** exhibited the micromolar activity only for pancreatic cancer cells PSN-1 (IC_50_ = 156.5 *μ*M/L) and for colorectal cancer cells SW480 (IC_50_ = 260 *μ*M/L). In the case of stilbenes** 5b** and** 6b** the growth inhibition effects occured only for pancreatic cancer cells PSN-1, (IC_50_ = 24.9 *μ*M/L) and (IC_50_ = 104.4 *μ*M/L) respectively. Stilbene** 7b** showed the micromolar activity only for breast cancer cells MDA-MB-231 (IC_50_ = 59.5 *μ*M/L).

## 4. Conclusions 

The methoxy stilbenes derivatives, such as combretastatin, possess antimimiotic and antivascular properties. Preclinical studies show that OXi4503 (combretastatin A1 diphosphate, CA1P) is more potent than other clinically evaluated vascular-disrupting agents. Considering the widespread use and importance of small molecules stilbenes, new and active stilbenes are looked for. In continuation of our studies on stilbenes, we synthesised seven novel 2-aminestilbenes with methoxy substitents in reactions of dinitrostilbenes with sodium azide. Due to unique features of stilbene derivatives, such as antitumor agents, we undertook the studies on the biological properties of new stilbene derivatives.

Using five cancer cell lines, we investigated the effects of 2-aminestilbenes with methoxy substitents on cell growth. Stilbene** 6b** exhibited the highest cytotoxic effect (of the investigated stilbenes) for cell line MDA-MB-231 and PSN1. In the case of cell line SW-480 cytotoxic effect occurred only at concentrations above 300 *μ*M for the stilbenes** 2b**,** 4b**,** 6b,** and** 7b**. For line A549 none of the stilbenes had a greater cytotoxic effect than 66%. For the cell line SKOV, the largest effect of cytotoxicity was for stilbenes** 3b** and** 6b**. When we calculated the half maximal inhibitory concentrations (IC_50_) for** 1b**–**7b** stilbenes, we concluded that for stilbenes** 1b**,** 3b** and** 4b** the growth inhibition effects were nonconverged. For other stilbenes we could calculate IC_50_, although not for all cancer cell lines.

The cytotoxicity of the obtained compounds can be important in narrowing the search for new, more cytotoxic molecules and can make these compounds the subject of great interest for further investigation.

## Supplementary Material

Experimental data e.g. yeld, melting point
Experimental NMR data
Mass Spectra data
IR spectra data
Computational data

## Figures and Tables

**Table 1:** The calculation angles between the *ortho*-nitro group in position 2 and ring of **1a–7a **and **1b–7b** stilbenes (the optimum structures of **1a–7a **and** 1b–7b **using the DFT B3LYP/6-311++G(2d, p) method were calculated.).

Compound	Angles (°)
**1a**	37.3/35.0
**2a**	32.5/32.8∗
**3a**	31.5/31.4
**4a**	33.2/32.7
**5a**	37.4/35.4
**6a**	34.5/34.5
**7a**	30.7/30.6
**1b**	32.5/17.1
**2b**	35.4/14.1
**3b**	35.5/14.0
**4b**	34.9/14.4
**5b**	34.6/14.7
**6b**	32.9/17.1
**7b**	23.8/22.3

*Data from ref. [10].

**Table 2:** Growth inhibition effects of **1b–7b** stilbenes on five cancer cell lines: breast cancer cells **MDA-MB-231**, pancreatic cancer cells **PSN-1**, colorectal cancer cells **SW480**, lung cancer cells **A549** and ovarian cancer cells **SKOV-3**.

Stilbene	Cancer cells									
	**MDA-MB-231**		**PSN-1**		**SW480**		**A549**		**SKOV-3**	
	IC_50_/ *μ*M	95% CI	IC_50_/ *μ*M	95% CI	IC_50_/ *μ*M	95% CI	IC_50_/ *μ*M	95% CI	IC_50_/ *μ*M	95% CI
**1b**	nonconverged	—	nonconverged	—	nonconverged	—	nonconverged	—	nonconverged	—
**2b**	nonconverged	—	156.5	111.5–219.8	260	220.5–306.5	nonconverged	—	nonconverged	—
**3b**	nonconverged	—	nonconverged	—	nonconverged	—	nonconverged	—	nonconverged	—
**4b**	nonconverged	—	nonconverged	—	nonconverged	—	nonconverged	—	nonconverged	—
**5b**	nonconverged	—	24.9	23.2–26.8	nonconverged	—	nonconverged	—	nonconverged	—
**6b**	nonconverged	—	104.4	57.3–190.0	nonconverged	—	nonconverged	—	nonconverged	—
**7b**	59.5	32.3–109.7	nonconverged	—	nonconverged	—	nonconverged	—	nonconverged	—
