# Supplementary material for: Cytotoxicity Studies of Novel Combretastatin and Pterostilbene Derivatives
Source: Biomed Res Int. 2014 Aug 3;2014:320895. doi: 10.1155/2014/320895 (PMC4137502; doi:10.1155/2014/320895)
Supplement: Supplementary file 1 — Experimental data e.g. yeld, melting point Experimental NMR data Mass Spectra data IR spectra data Computational data [file 320895.f1.pdf]

Cytotoxicity studies of novel Combretastatin and Pterostilben derivatives

Joanna Jakubowska[a], Justyna Mikuła-Pietrasik[b] Krzysztof Książek[b] and Hanna Krawczyk *[a]

[a]Department of Organic Chemistry, Faculty of ChemistryWarsaw University of Technology, Noakowskiego 3,00-664 Warsaw, Poland,

Fax: (+)48 22 628 27 41 ,E-mail: *hkraw@ch.pw.edu.pl*

[b] Department of Pathophysiology, Laboratory of Gerontology, Poznań University of Medical Sciences. ul. Święcickiego 6, 60-781 Poznań, Poland

Experimental. All stilbenes (**1b-7b**) were prepared in our laboratory. Sodium azide was obtained from Sigma-Aldrich Corp.
The data for compounds (**1b**) and (**2b**) in [1].
Compound (3b) -stirred at 120 °C for 20 h, yield 82% (436 mg; 1.45 mmol), bright red powder, mp 137^{0}C;
Compound (4b)- stirred at 120 ^{0}C for 20 h, yield 80% (436 mg; 1.36 mmol), dark red powder, mp 117^{0}C ;
Compound (5b)- stirred at 120 ^{0}C for 20 h, yield 81% (436 mg; 1.51 mmol), dark orange powder, mp 105^{0}C;
Compound (6b)- stirred at 120 ^{0}C for 20 h, yield 78% (436 mg; 1.28 mmol), yellow powder, mp 153^{0}C.
Compound (7b)- stirred at 120 ^{0}C for 20 h, yield 75% (479 mg; 1.45 mmol), red powder, mp 144^{0}C ;

Mass Spectra
The measurement of mass spectra were carried out with ESI-TOF MS Mariner (Perseptive Biosystem).
The data for compounds (**1b**) and (**2b**) in [1].
HRMS of (**3b**)(ESI, MeOH/ $CHCl_3$) 323.1008 calcd for $C_{16}H_{16}N_2O_4Na$ (M^++Na) found 323.0996;
HRMS of (**4b**)(ESI, MeOH/ $CHCl_3$) 323.1008 calcd for $C_{16}H_{16}N_2O_4Na$ (M^++Na) found 323.0996;
HRMS of (**5b**)(ESI, MeOH/ $CHCl_3$) 323.1008 calcd for $C_{16}H_{16}N_2O_4Na$ (M^++Na) found 323.1007;
HRMS of (**6b**)(ESI, MeOH/ $CHCl_3$) 323.1008 calcd for $C_{16}H_{16}N_2O_4Na$ (M^++Na) found 323.1005;
HRMS of (**7b**)(ESI, MeOH/ $CHCl_3$) 353.1113 calcd for $C_{17}H_{18}N_2O_5Na$ (M^++Na) found 353.1111.

IR spectra
The IR spectra were recorded at ambient temperature on a Perkin Elmer System 2000, using the technique of Attenuated Total Reflectance (ATR).
The data for compounds (**1b**) and (**2b**) in [1].
IR of (**3b**) ν(cm^{-1}): 3439.8, 3351.6, 2959.1, 2836.7, 1639.0, 1583.4, 1505.9, 1463.8, 1334.4, 1136.1 (the whole spectrum is on the following pages).
IR of (**4b**) ν(cm^{-1}): 3496.9, 3403.3, 2932.4, 2835.0, 1604.8, 1575.6, 1500.6, 1447.1, 1339.8, 1156.6(the whole spectrum is on the following pages)
IR of (**5b**) ν(cm^{-1}): 3473.8, 3383.9, 2936.5, 2834.0, 1639.9, 1580.0, 1495.1, 1425.5, 1318.3, 1215.4 (the whole spectrum is on the following pages).
IR of (**6b**)ν(cm^{-1}): 3417.8, 3343.1, 2925.7, 2844.0, 1644.1, 1593.4, 1520.1, 1455.4, 1341.4, 1148.8 (the whole spectrum is on the following pages).
IR of (**7b**) ν(cm^{-1}): 3432.8, 3352.9, 2942.9, 2839.0, 1639.8, 1581.9, 1505.2, 1420.5, 1326.3, 1122.5 (the whole spectrum is on the following pages).

Experimental NMR

All the spectra were recorded using a Varian VNMRS spectrometer operating at 11.7 T magnetic field. Measurements were performed for ca. 1.0M solutions of all the compounds in DMSO-d_6. The residual signals of DMSO-d_6 (2.54ppm) in ^{1}H NMR and the DMSO-d_6 signal (40.45 ppm) in ^{13}C NMR spectra were used as the chemical shift references. All the proton spectra were recorded using the standard spectrometer software and parameters set: acquisition time 3 s, pulse angle 30°. The standard measurement parameter set for ^{13}C spectra was: pulse width 7 μs (the 90° pulse width was 12.5 μs), acquisition time 1 s, spectral width 200 ppm, 1000 scans of 32 K data point were accumulated and after zero-filling to 64 K; and the FID signals were subjected to Fourier transformation after applying a 1 Hz line broadening. The ^{1}H–^{13}Cgs-HSQC and ^{1}H–^{13}Cgs-HMBC spectra were also recorded using the standard Varian software.

Table 1. Experimental ^{1}H chemical shifts and coupling constants of (**1b**)-(**7b**) in DMSO-d_6.

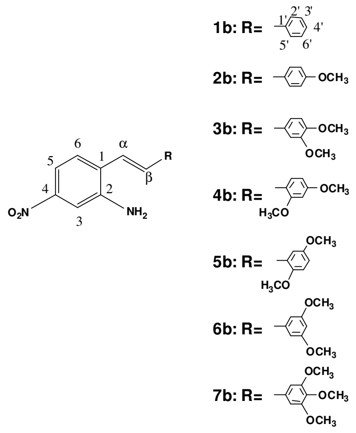

**Compound**	**^{1}H NMR [ppm] and J [Hz]**										
	**2(NH_2)**	**3**	**5**	**6**	**α**	**β**	**2’**	**3’**	**4’**	**5’**	**6’**
**(1b)**	6.10;bs	7.58; d<br>$J_{3,5}$=2.5	7.40; dd<br>$J_{5,6}$=8.5	7.72; d	7.28; AB	7.48; AB $J_{\alpha,\beta}$=<br>16.3	7.73; d	7.43; d<br>$J_{2',3'}$= 7.5	7.33; t<br>$J_{3',4'}$ = 7.5<br>$J_{4',5'}$ = 7.5	7.42; d<br>$J_{4',5'}$=7.5	7.73;d<br>$J_{5',6'}$= 7.5
**(2b)**	6.02;bs	7.55<br>$J_{3,5}$=2.0	7.40; dd<br>$J_{5,6}$=8.5	7.69; d	7.24; AB	7.32 AB<br>$J_{\alpha,\beta}$=16.0	7.67; d	7.00; d<br>$J_{2',3'}$= 8.5	3.83; s(CH_3)	7.00; d	7.67; d<br>$J_{5',6'}$= 8.5
**(3b)**	6.07;bs	7.56; d<br>$J_{3,5}$=2.5	7.40; dd<br>$J_{5,6}$=8.5	7.69; d	7.32; AB	7.22; AB<br>$J_{\alpha,\beta}$= 16.0	7.41; d<br>$J_{2',6'}$=2.0	3.88;s(CH_3)	3.82; s(CH_3)	7.01; d<br>$J_{5',6'}$=8.5	7.21; dd
**(4b)**	5.96; bs	7.56; d<br>$J_{3,5}$=2.5	7.41; dd<br>$J_{5,6}$=8.5	7.60; d	7.26; AB	7.38; AB<br>$J_{\alpha,\beta}$= 16.0	3.89; s(CH_3)	6.64; d<br>$J_{3',5'}$=2.0	3.84; s(CH_3)	6.63; dd<br>$J_{5',6'}$=9.5	7.79; d
**(5b)**	6.08;bs	7.57;d<br>$J_{3,5}$=2.5	7.41<br>$J_{5,6}$=8.5	7.64; d	7.41; AB	7.43; AB<br>$J_{\alpha,\beta}$= 16.0	3.84;s(CH_3)	7.01<br>$J_{3',4'}$=9.0	6.92<br>$J_{4',6'}$=3.0	3.82;s(CH_3)	7.46; d
**(6b)**	6.13;bs	7.56;d<br>$J_{3,5}$=2.5	7.40<br>$J_{5,6}$=8.5	7.71; d	7.45; AB	7.22; AB<br>$J_{\alpha,\beta}$= 16.0	6.93; d<br>$J_{2',4'}$=2.5	3.83;s(CH_3)	6.49; t<br>$J_{4',6'}$=2.5	3.83;s(CH_3)	6.93; d
**(7b)**	6.11; bs	7.57; d<br>$J_{3,5}$=2.5	7.41<br>$J_{5,6}$=8.5	7.69; d	7.38; AB	7.23; AB<br>$J_{\alpha,\beta}$= 16.0	7.06;s	3.88;s(CH_3)	3.73; s(CH_3)	3.88;s(CH_3)	7.06;s

Table 2. Experimental ^{13}C chemical shifts of (**1b**)-(**7b**) in DMSO-d_6 (the residual signal 40.45ppm).

Compound	^{13}C NMR [ppm]														
	**1**	**2**	**3**	**4**	**5**	**6**	**α**	**β**	**1'**	**2'**	**3'**	**4'**	**5'**	**6'**	**substituent**
**(1b)**	129.55	148.07	110.03	148.14	111.48	127.16	123.43	132.21	138.03	127.93	129.59	128.96	129.59	127.93	-
**(2b)**	128.87	147.74	109.90	147.74	111.61	126.70	121.03	132.02	130.74	129.36	115.09	160.28	115.09	129.36	56.16 (C4'-OCH_3)
**(3b)**	128.75	147.70	109.84	147.72	111.57	126.61	121.00	132.38	130.94	110.43	149.86	150.10	112.59	121.83	56.52 (C3'-OCH_3); 56.45 (C4'-OCH_3)
**(4b)**	129.50	147.63	109.85	147.64	111.81	126.56	121.06	126.32	119.33	158.78	99.16	161.78	106.75	128.41	56.59 (C2'-OCH_3); 56.28 (C4'-OCH_3)
**(5b)**	128.56	147.99	109.91	148.05	111.59	127.00	123.68	125.96	127.07	151.93	113.56	115.56	154.27	112.38	57.03 (C2'-OCH_3); 56.47 (C5'-OCH_3)
**(6b)**	127.95	148.06	109.97	148.15	111.39	127.11	123.73	132.24	139.95	105.97	161.55	101.15	161.55	105.97	56.19 (C3'-OCH_3; C5'-OCH_3)
**(7b)**	128.28	147.87	109.91	147.97	111.51	126.81	122.55	132.42	133.66	105.42	153.96	138.66	153.96	105.42	56.87 (C3',C5'-OCH3); 61.03 (C4'-OCH3)

Computational aspects

The optimum ground-state geometry for **1a, 3a-7a** and for **1b-7b** compounds were calculated using the density functional theory (DFT). In calculation the B3LYP functional and 6-311G 6-311++g (2d,p) basis set was employed and the continuum model (PCM; Gaussian 03W) [2,3] was used in order to simulate the effects of the solvent. All the calculations were performed on a server equipped with a 16 quad-core XEON (R) CPU E7310 processor operating at 1.60 GHz. The operating system was Open SUSE 10.3. The data for compounds (**2a**) in [1].

The calculated coordinates of (**1a**)(the part of calculated log file)

```
Standard orientation:
 ---------------------------------------------------------------------
 Center     Atomic     Atomic              Coordinates (Angstroms)
 Number     Number      Type              X           Y           Z
 ---------------------------------------------------------------------
    1          6             0        5.303617    0.856801   -0.193348
    2          6             0        3.926301    0.711714   -0.217677
    3          6             0        3.332495   -0.538558    0.028813
    4          6             0        4.173651   -1.630923    0.295690
```

5	6	0	5.554063	-1.484003	0.321018
6	6	0	6.124113	-0.238972	0.077623
7	6	0	-0.990360	-1.424789	-0.227623
8	6	0	-2.332700	-1.727329	-0.177505
9	6	0	-3.254855	-0.693165	-0.030038
10	6	0	-2.842523	0.621250	0.062178
11	6	0	-1.487743	0.901203	-0.020287
12	6	0	-0.497097	-0.103292	-0.149584
13	6	0	1.892161	-0.764213	0.015947
14	6	0	0.933324	0.168241	-0.158500
15	7	0	-1.156846	2.335420	0.054687
16	8	0	-0.232524	2.762051	-0.629763
17	8	0	-1.850890	3.035188	0.784101
18	7	0	-4.683840	-0.995019	0.022837
19	8	0	-5.469126	-0.058909	0.147365
20	8	0	-5.022621	-2.172629	-0.058945
21	1	0	5.744257	1.827977	-0.385001
22	1	0	3.311055	1.575998	-0.434358
23	1	0	3.732819	-2.603998	0.483207
24	1	0	6.184089	-2.340244	0.530396
25	1	0	7.200951	-0.119979	0.093822
26	1	0	-0.291108	-2.239676	-0.350606
27	1	0	-2.674783	-2.749132	-0.257546
28	1	0	-3.559852	1.418032	0.185513
29	1	0	1.608124	-1.798323	0.183989
30	1	0	1.223860	1.197149	-0.294394

Visualization of calculated geometry of (**1a**):

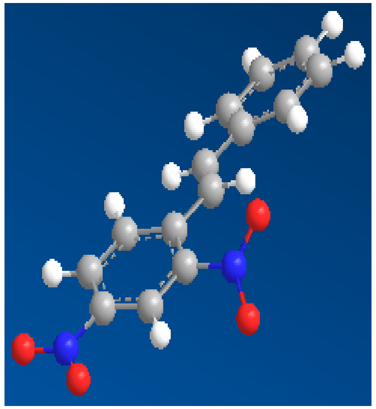

The calculated coordinates of (**3a**)(the part of calculated log file)

Standard orientation:

Center Number	Atomic Number	Atomic Type	Coordinates (Angstroms) X	Y	Z
1	6	0	3.846662	1.324327	-0.366356
2	6	0	2.483416	1.081364	-0.331414
3	6	0	1.978206	-0.170551	0.046503
4	6	0	2.906319	-1.175202	0.374902

5	6	0	4.268834	-0.950906	0.342300
6	6	0	4.759027	0.320786	-0.022942
7	6	0	-2.265556	-1.368250	-0.165999
8	6	0	-3.584540	-1.761159	-0.164056
9	6	0	-4.582996	-0.790677	-0.074889
10	6	0	-4.265037	0.550514	0.014233
11	6	0	-2.929218	0.918358	0.044335
12	6	0	-1.868508	-0.016428	-0.050123
13	6	0	0.561884	-0.486184	0.112475
14	6	0	-0.467694	0.359823	-0.107089
15	8	0	5.122690	-1.949339	0.741031
16	6	0	5.884945	-2.564829	-0.309872
17	7	0	-2.686530	2.359692	0.212884
18	8	0	-1.690094	2.716292	0.833825
19	8	0	-3.515748	3.132836	-0.257436
20	7	0	-5.986187	-1.189317	-0.084130
21	8	0	-6.840194	-0.308684	-0.008312
22	8	0	-6.240115	-2.388972	-0.166910
23	8	0	6.101723	0.476461	-0.001126
24	6	0	6.651557	1.747992	-0.353656
25	1	0	4.198502	2.304806	-0.655419
26	1	0	1.811954	1.885631	-0.603816
27	1	0	2.562065	-2.159686	0.672366
28	1	0	-1.505337	-2.126576	-0.291629
29	1	0	-3.853333	-2.804435	-0.250179
30	1	0	-5.039606	1.299147	0.078379
31	1	0	0.347986	-1.511396	0.399777
32	1	0	-0.255535	1.397471	-0.316562
33	1	0	6.501552	-3.323503	0.168604
34	1	0	6.522635	-1.835814	-0.811667
35	1	0	5.217276	-3.039537	-1.034495
36	1	0	7.729122	1.635966	-0.262444
37	1	0	6.396620	2.015116	-1.382080
38	1	0	6.305228	2.527551	0.329191

Visualization of calculated geometry of (**3a**):

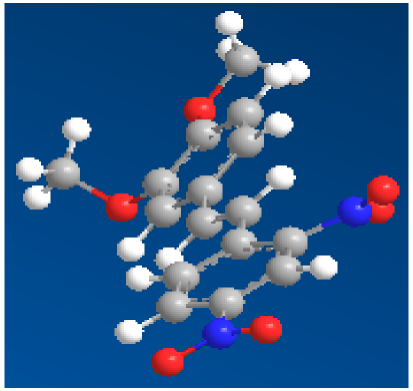

The calculated coordinates of (**4a**)(the part of calculated log file)

Standard orientation:

Center Number	Atomic Number	Atomic Type	Coordinates (Angstroms) X	Y	Z
1	6	0	-4.097362	-1.366608	-0.280757
2	6	0	-2.740088	-1.105044	-0.234817
3	6	0	-2.218256	0.180731	-0.035798
4	6	0	-3.156123	1.242026	0.120222
5	6	0	-4.519976	0.996220	0.075013
6	6	0	-4.994490	-0.304002	-0.124049
7	6	0	2.055018	1.246809	-0.209397
8	6	0	3.382801	1.600639	-0.238323
9	6	0	4.356757	0.606463	-0.120098
10	6	0	4.000320	-0.719841	0.028283
11	6	0	2.654936	-1.048102	0.088917
12	6	0	1.615869	-0.088395	-0.034858
13	6	0	-0.802306	0.468555	0.012279
14	6	0	0.209799	-0.429478	-0.058433
15	7	0	2.379991	-2.474618	0.317665
16	8	0	3.170207	-3.287797	-0.154073
17	8	0	1.398503	-2.783611	0.987545
18	8	0	-6.341275	-0.430358	-0.150613
19	6	0	-6.907831	-1.727126	-0.354030
20	7	0	5.767447	0.963550	-0.157703
21	8	0	6.599068	0.063261	-0.051475
22	8	0	6.055531	2.151686	-0.295203
23	8	0	-2.633397	2.477426	0.311508
24	6	0	-3.515532	3.589821	0.463908
25	1	0	-4.442197	-2.378186	-0.437211
26	1	0	-2.059286	-1.936963	-0.364361
27	1	0	-5.246009	1.787685	0.190864
28	1	0	1.318734	2.025358	-0.348997
29	1	0	3.680090	2.631540	-0.368715
30	1	0	4.753927	-1.487453	0.113055
31	1	0	-0.569208	1.517522	0.141419
32	1	0	-0.026433	-1.480526	-0.117063
33	1	0	-7.985225	-1.581376	-0.342724
34	1	0	-6.604310	-2.140618	-1.318613

35	1	0	-6.622467	-2.410111	0.449571
36	1	0	-2.873333	4.457559	0.594102
37	1	0	-4.150758	3.469137	1.344743
38	1	0	-4.135912	3.723053	-0.425680

Visualization of calculated geometry of (**4a**):

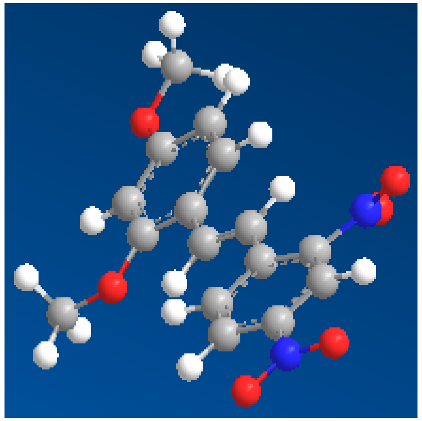

The calculated coordinates of (**5a**)(the part of calculated log file)

Standard orientation:

Center Number	Atomic Number	Atomic Type	Coordinates (Angstroms) X	Y	Z
1	6	0	4.293359	-0.961203	-0.075100
2	6	0	2.916163	-0.783904	-0.109572
3	6	0	2.330842	0.483314	-0.023778
4	6	0	3.184943	1.610806	0.100065
5	6	0	4.563223	1.430258	0.133174
6	6	0	5.119880	0.156050	0.047248
7	6	0	-2.006877	1.278393	-0.284099
8	6	0	-3.354765	1.553541	-0.260864
9	6	0	-4.258666	0.508296	-0.071754
10	6	0	-3.818204	-0.791163	0.083541
11	6	0	-2.457271	-1.046711	0.025418
12	6	0	-1.484940	-0.027926	-0.139123
13	6	0	0.892343	0.689943	-0.063648
14	6	0	-0.053080	-0.274314	-0.110351
15	8	0	2.578476	2.826889	0.183832
16	6	0	3.392097	3.993289	0.286896
17	8	0	4.736505	-2.251069	-0.169362
18	6	0	6.141661	-2.483248	-0.128927
19	7	0	-2.097453	-2.468590	0.156644
20	8	0	-1.161872	-2.900916	-0.509599
21	8	0	-2.779529	-3.155412	0.910184
22	7	0	-5.691498	0.781795	-0.043301
23	8	0	-6.459131	-0.163450	0.122894
24	8	0	-6.054629	1.947118	-0.185791
25	1	0	2.299088	-1.666965	-0.212566
26	1	0	5.228185	2.276820	0.227641
27	1	0	6.196155	0.057898	0.076049
28	1	0	-1.324320	2.102802	-0.431085

29	1	0	-3.717402	2.563845	-0.386675
30	1	0	-4.519872	-1.597358	0.234329
31	1	0	0.597004	1.730307	-0.042115
32	1	0	0.250176	-1.308823	-0.109806
33	1	0	2.700662	4.831569	0.330855
34	1	0	4.040765	4.100238	-0.586599
35	1	0	3.998419	3.972492	1.196326
36	1	0	6.267280	-3.560475	-0.211807
37	1	0	6.649692	-1.993824	-0.964893
38	1	0	6.574021	-2.138743	0.814931

Visualization of calculated geometry of (**5a**):

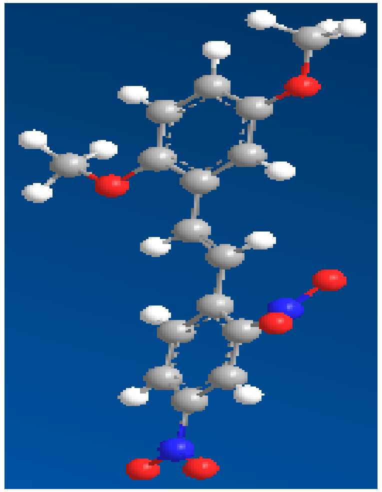

The calculated coordinates of (**6a**)(the part of calculated log file)

Standard orientation:

Center Number	Atomic Number	Atomic Type	Coordinates (Angstroms) X	Y	Z
1	6	0	3.988119	0.949302	-0.382115
2	6	0	2.607645	0.788115	-0.365249
3	6	0	2.055873	-0.455517	-0.033123
4	6	0	2.904275	-1.525850	0.272572
5	6	0	4.286548	-1.353208	0.253554
6	6	0	4.843507	-0.115393	-0.072390
7	6	0	-2.247460	-1.391043	-0.414998
8	6	0	-3.589025	-1.709535	-0.440635
9	6	0	-4.527798	-0.716461	-0.165566
10	6	0	-4.137861	0.574819	0.140504
11	6	0	-2.782834	0.856345	0.192145
12	6	0	-1.781912	-0.100702	-0.090722
13	6	0	0.614805	-0.701600	0.014943
14	6	0	-0.359325	0.214641	-0.134705
15	8	0	4.434389	2.194318	-0.717602
16	6	0	5.841157	2.437975	-0.746468
17	7	0	-5.950301	-1.034099	-0.205081
18	8	0	-6.755512	-0.138771	0.042894
19	8	0	-6.277003	-2.185222	-0.486631
20	8	0	5.026161	-2.454767	0.569084
21	6	0	6.450140	-2.348490	0.567005

22	7	0	-2.439742	2.226526	0.596712
23	8	0	-1.438619	2.396530	1.285370
24	8	0	-3.191680	3.130993	0.245173
25	1	0	1.988896	1.640081	-0.624466
26	1	0	2.495597	-2.498458	0.529920
27	1	0	5.912663	0.018808	-0.088611
28	1	0	-1.527673	-2.156905	-0.684435
29	1	0	-3.910591	-2.713679	-0.692168
30	1	0	-4.869056	1.342619	0.359024
31	1	0	0.342391	-1.733827	0.228960
32	1	0	-0.096949	1.254381	-0.281549
33	1	0	5.953344	3.482078	-1.030351
34	1	0	6.290277	2.278842	0.237611
35	1	0	6.337261	1.805488	-1.487681
36	1	0	6.819875	-3.332621	0.846329
37	1	0	6.791907	-1.610473	1.297750
38	1	0	6.825348	-2.088355	-0.426578

Visualization of calculated geometry of (**6a**):

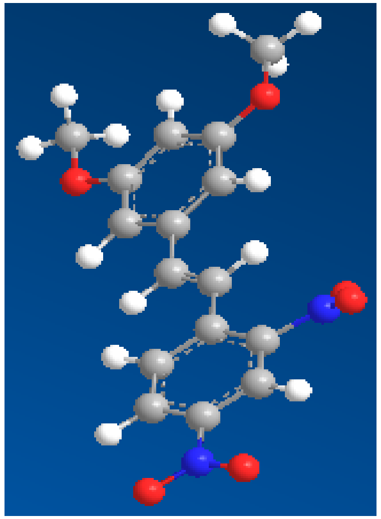

The calculated coordinates of (**7a**)(the part of calculated log file)

Standard orientation:

Center Number	Atomic Number	Atomic Type	Coordinates (Angstroms) X	Y	Z
1	6	0	3.558551	0.918697	-0.336628
2	6	0	2.182261	0.748763	-0.298955
3	6	0	1.626872	-0.503571	0.019618
4	6	0	2.481845	-1.576944	0.284646
5	6	0	3.862713	-1.413650	0.250389
6	6	0	4.416527	-0.167583	-0.048099
7	6	0	-2.675182	-1.463046	-0.243576
8	6	0	-4.012963	-1.790396	-0.244057
9	6	0	-4.958307	-0.780505	-0.071536
10	6	0	-4.573277	0.534597	0.100960
11	6	0	-3.220141	0.833249	0.130155
12	6	0	-2.211139	-0.144459	-0.042950
13	6	0	0.191544	-0.744457	0.080947

14	6	0	-0.790260	0.162275	-0.096106
15	8	0	4.663046	-2.477642	0.582878
16	6	0	5.400639	-3.058478	-0.504934
17	7	0	-2.900874	2.247707	0.385197
18	8	0	-1.870397	2.513120	0.995571
19	8	0	-3.703088	3.088620	-0.008020
20	8	0	5.773017	-0.018696	-0.115500
21	6	0	6.376025	0.611125	1.029220
22	8	0	4.185263	2.084516	-0.637818
23	6	0	3.385722	3.233484	-0.914797
24	7	0	-6.381887	-1.108995	-0.081109
25	8	0	-7.187944	-0.195033	0.072990
26	8	0	-6.695865	-2.285394	-0.242911
27	1	0	1.535476	1.583497	-0.523083
28	1	0	2.082562	-2.553543	0.531382
29	1	0	-1.955160	-2.246869	-0.433556
30	1	0	-4.335348	-2.810825	-0.395355
31	1	0	-5.308546	1.314106	0.228489
32	1	0	-0.073719	-1.768285	0.326017
33	1	0	-0.527324	1.195783	-0.264426
34	1	0	5.976755	-3.876273	-0.075901
35	1	0	6.073649	-2.327754	-0.954766
36	1	0	4.714378	-3.452228	-1.260209
37	1	0	7.438829	0.685897	0.807086
38	1	0	2.748160	3.070607	-1.787842
39	1	0	6.228400	-0.003221	1.920730
40	1	0	4.087979	4.036932	-1.124059
41	1	0	5.961748	1.608347	1.185320
42	1	0	2.770652	3.502303	-0.051757

Visualization of calculated geometry of (**7a**):

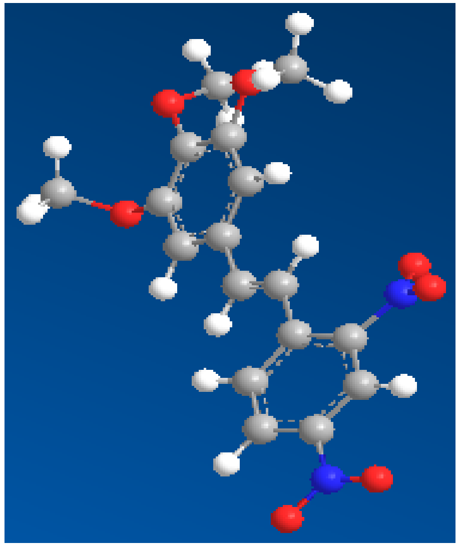

The calculated coordinates of (**1b**)(the part of calculated log file)

Standard orientation:

Center Number	Atomic Number	Atomic Type	Coordinates (Angstroms) X	Y	Z
1	6	0	-5.203113	1.065976	0.100372
2	6	0	-3.822770	0.940139	0.111510
3	6	0	-3.213248	-0.320598	-0.007049
4	6	0	-4.046526	-1.443464	-0.133945
5	6	0	-5.429974	-1.317235	-0.144679
6	6	0	-6.015128	-0.061013	-0.028055
7	6	0	1.175721	-1.105143	0.147155
8	6	0	2.532872	-1.350586	0.114226
9	6	0	3.387832	-0.256833	0.007156
10	6	0	2.909864	1.041529	-0.051787
11	6	0	1.534798	1.288620	-0.017126
12	6	0	0.631880	0.189350	0.068572
13	6	0	-1.766530	-0.527413	-0.004607
14	6	0	-0.809437	0.417501	0.068000
15	7	0	1.092219	2.598459	-0.140845
16	7	0	4.829242	-0.478278	-0.030455
17	8	0	5.570550	0.500132	-0.130617
18	8	0	5.242504	-1.636253	0.039046
19	1	0	-5.651973	2.048092	0.193736
20	1	0	-3.217364	1.832087	0.217139
21	1	0	-3.596854	-2.426331	-0.225555
22	1	0	-6.050488	-2.200185	-0.244126
23	1	0	-7.093713	0.042728	-0.035163
24	1	0	0.508460	-1.951509	0.241022
25	1	0	2.928210	-2.353321	0.173004
26	1	0	3.608200	1.864159	-0.123882
27	1	0	-1.471899	-1.569583	-0.081152
28	1	0	-1.133770	1.450082	0.108357
29	1	0	0.220859	2.841412	0.307816
30	1	0	1.798223	3.305101	0.022002

Visualization of calculated geometry of (**1b**):

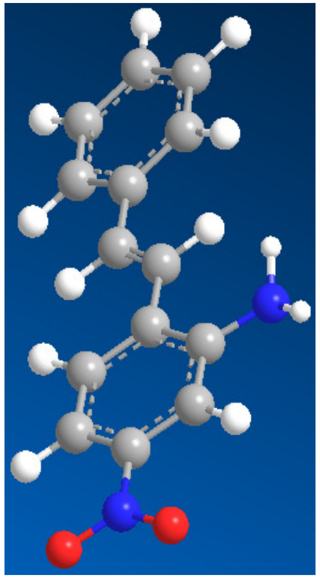

The calculated coordinates of (**2b**)(the part of calculated log file):

Standard orientation:

Center Number	Atomic Number	Atomic Type	Coordinates (Angstroms) X	Y	Z
1	6	0	4.351239	0.773658	0.392911
2	6	0	2.966936	0.688084	0.356211
3	6	0	2.310221	-0.454863	-0.120220
4	6	0	3.114016	-1.524818	-0.553723
5	6	0	4.493786	-1.456134	-0.523601
6	6	0	5.127086	-0.302661	-0.050682
7	6	0	-2.087890	-1.103385	0.275835
8	6	0	-3.455742	-1.299014	0.292091
9	6	0	-4.273860	-0.193888	0.075921
10	6	0	-3.753233	1.069461	-0.167390
11	6	0	-2.372186	1.260659	-0.194928
12	6	0	-1.508934	0.154372	0.042828
13	6	0	0.859982	-0.590948	-0.185299
14	6	0	-0.064409	0.353999	0.074919
15	7	0	-1.855569	2.533224	-0.408141
16	7	0	-5.721269	-0.363527	0.099862
17	8	0	-6.431181	0.627901	-0.077319
18	8	0	-6.174450	-1.492531	0.294893
19	8	0	6.486270	-0.322056	-0.055713
20	6	0	7.187231	0.828336	0.415261
21	1	0	4.813333	1.675093	0.770642
22	1	0	2.394330	1.534453	0.716167
23	1	0	2.638379	-2.426718	-0.923859
24	1	0	5.101884	-2.286523	-0.861801
25	1	0	-1.440066	-1.944992	0.485834
26	1	0	-3.886411	-2.269490	0.487229
27	1	0	-4.424232	1.897868	-0.348975
28	1	0	0.516077	-1.572187	-0.502549
29	1	0	0.266131	1.354196	0.336544
30	1	0	-0.982968	2.583577	-0.916157
31	1	0	-2.521597	3.213359	-0.752459
32	1	0	8.244038	0.590648	0.318538
33	1	0	6.954527	1.707953	-0.190724
34	1	0	6.953876	1.031208	1.463812

Visualization of calculated geometry of (**2b**):

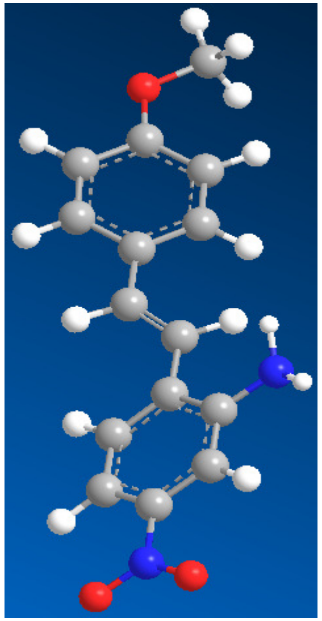

The calculated coordinates of (**3b**)(the part of calculated log file):

Standard orientation:

Center Number	Atomic Number	Atomic Type	Coordinates (Angstroms) X	Y	Z
1	6	0	-3.663511	1.408286	-0.455690
2	6	0	-2.290899	1.217234	-0.391057
3	6	0	-1.743248	-0.045804	-0.135819
4	6	0	-2.638248	-1.112364	0.053873
5	6	0	-4.009038	-0.938823	-0.003077
6	6	0	-4.542400	0.337751	-0.269941
7	6	0	2.610054	-0.929201	-0.565211
8	6	0	3.959984	-1.223507	-0.592681
9	6	0	4.843522	-0.271256	-0.093006
10	6	0	4.403787	0.934004	0.436034
11	6	0	3.039730	1.222239	0.473036
12	6	0	2.112241	0.277535	-0.048738
13	6	0	-0.310675	-0.311108	-0.060427
14	6	0	0.687978	0.592698	-0.070274
15	7	0	2.604424	2.442413	0.975822
16	7	0	6.275415	-0.544592	-0.120360
17	8	0	7.045277	0.315375	0.310585
18	8	0	6.655188	-1.625742	-0.572487
19	8	0	-5.894060	0.425881	-0.345312
20	6	0	-6.479281	1.701758	-0.605319
21	8	0	-4.827901	-2.035563	0.128233
22	6	0	-5.574111	-2.101584	1.353088
23	1	0	-4.046322	2.397097	-0.667137
24	1	0	-1.645824	2.070859	-0.557962
25	1	0	-2.263177	-2.111005	0.249782
26	1	0	1.914174	-1.640497	-0.991391
27	1	0	4.329197	-2.149331	-1.007292
28	1	0	5.123234	1.638287	0.830787
29	1	0	-0.052697	-1.362778	0.033792
30	1	0	0.440077	1.649144	-0.100430
31	1	0	1.716618	2.436558	1.459406
32	1	0	3.300788	2.966355	1.490892
33	1	0	-7.553872	1.535468	-0.616879
34	1	0	-6.228449	2.418131	0.181247
35	1	0	-6.159923	2.091693	-1.575189

36	1	0	-6.158192	-3.018934	1.303027
37	1	0	-6.242544	-1.245224	1.452540
38	1	0	-4.895308	-2.147316	2.209679

Visualization of calculated geometry of (**3b**):

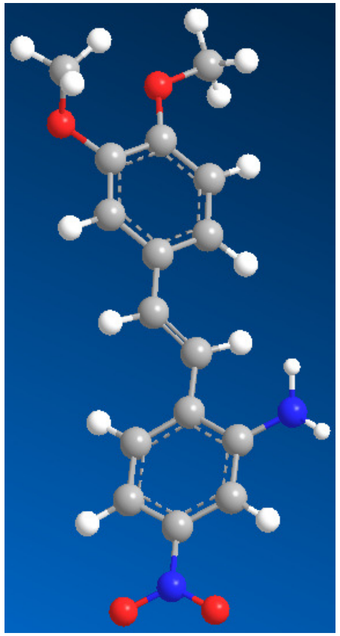

The calculated coordinates of (**4b**)(the part of calculated log file):

Standard orientation:

Center Number	Atomic Number	Atomic Type	Coordinates (Angstroms) X	Y	Z
1	6	0	3.940264	-1.427643	-0.471715
2	6	0	2.570685	-1.206791	-0.430035
3	6	0	2.009664	0.026982	-0.089112
4	6	0	2.910993	1.083849	0.208444
5	6	0	4.283307	0.882509	0.169633
6	6	0	4.800955	-0.372263	-0.166700
7	6	0	-2.346219	0.758324	-0.655939
8	6	0	-3.701787	1.016443	-0.726149
9	6	0	-4.572186	0.083527	-0.169451
10	6	0	-4.112672	-1.068495	0.454582
11	6	0	-2.743653	-1.320508	0.532096
12	6	0	-1.828129	-0.393665	-0.042077
13	6	0	0.574489	0.266213	-0.049998
14	6	0	-0.396973	-0.668701	-0.009177
15	7	0	-2.289716	-2.490495	1.130029
16	8	0	6.157526	-0.457418	-0.173231
17	6	0	6.759234	-1.709789	-0.499841
18	8	0	2.345090	2.275193	0.537805
19	6	0	3.192805	3.384792	0.826963
20	7	0	-6.007788	0.319587	-0.238270

21	8	0	-6.765428	-0.520089	0.251929
22	8	0	-6.405934	1.350813	-0.783293
23	1	0	4.315674	-2.401315	-0.750964
24	1	0	1.913519	-2.025937	-0.695740
25	1	0	4.981662	1.674528	0.398298
26	1	0	-1.659623	1.452284	-1.123619
27	1	0	-4.085232	1.899644	-1.214419
28	1	0	-4.822210	-1.758778	0.889966
29	1	0	0.286344	1.310813	-0.025960
30	1	0	-0.118396	-1.715776	0.056609
31	1	0	-1.406831	-2.430085	1.618634
32	1	0	-2.981004	-2.987339	1.677570
33	1	0	7.832660	-1.546740	-0.437177
34	1	0	6.465609	-2.485629	0.211875
35	1	0	6.496268	-2.021245	-1.513981
36	1	0	2.526033	4.215287	1.047293
37	1	0	3.819861	3.637383	-0.032065
38	1	0	3.823507	3.182579	1.696533

Visualization of calculated geometry of (**4b**):

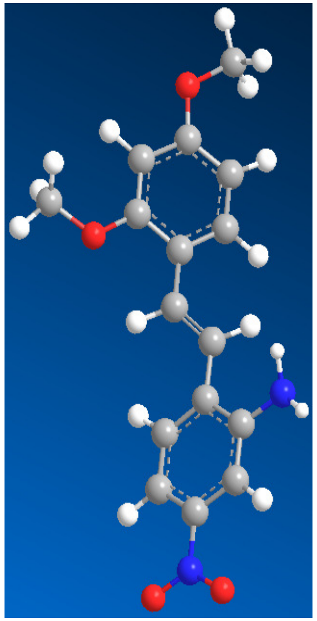

The calculated coordinates of (**5b**)(the part of calculated log file):

Standard orientation:

Center Number	Atomic Number	Atomic Type	Coordinates (Angstroms) X	Y	Z
1	6	0	4.135017	-1.064943	-0.293089
2	6	0	2.750722	-0.917464	-0.292880
3	6	0	2.139837	0.312143	-0.042339
4	6	0	2.970325	1.434176	0.202900
5	6	0	4.351717	1.286629	0.198112
6	6	0	4.939015	0.044621	-0.046418
7	6	0	-2.246152	0.746651	-0.720681
8	6	0	-3.613608	0.919538	-0.823352
9	6	0	-4.434686	-0.005015	-0.184849
10	6	0	-3.919921	-1.063777	0.548983
11	6	0	-2.538755	-1.228682	0.659610
12	6	0	-1.672916	-0.310368	0.003064

13	6	0	0.688909	0.480675	-0.041446
14	6	0	-0.227155	-0.500112	0.065657
15	7	0	-2.027379	-2.307017	1.369333
16	7	0	-5.883290	0.141076	-0.284140
17	8	0	-6.596314	-0.689765	0.279877
18	8	0	-6.331031	1.091015	-0.927357
19	8	0	2.332386	2.616948	0.448380
20	6	0	3.121432	3.784641	0.651075
21	8	0	4.603141	-2.324465	-0.556204
22	6	0	6.012512	-2.525529	-0.566787
23	1	0	2.148631	-1.789336	-0.516846
24	1	0	4.997581	2.132321	0.387634
25	1	0	6.017494	-0.029429	-0.045320
26	1	0	-1.593672	1.431418	-1.247400
27	1	0	-4.040798	1.729233	-1.395364
28	1	0	-4.594317	-1.749157	1.043954
29	1	0	0.348606	1.507444	-0.103794
30	1	0	0.109503	-1.521272	0.216504
31	1	0	-1.152282	-2.155745	1.852133
32	1	0	-2.694839	-2.792656	1.955164
33	1	0	2.414534	4.598421	0.797522
34	1	0	3.746650	3.997717	-0.220627
35	1	0	3.752406	3.687904	1.539134
36	1	0	6.161832	-3.580632	-0.785807
37	1	0	6.494985	-1.922705	-1.341895
38	1	0	6.455144	-2.290908	0.405731

Visualization of calculated geometry of (**5b**):

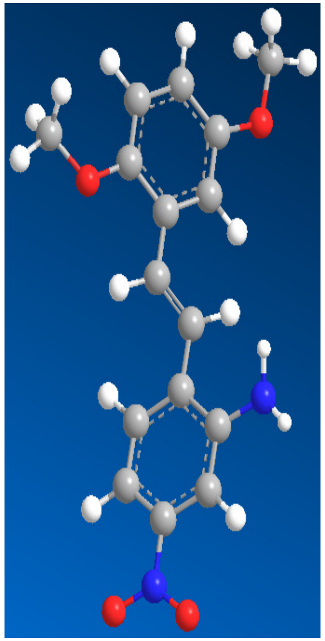

The calculated coordinates of (**6b**)(the part of calculated log file):

Standard orientation:

Center Number	Atomic Number	Atomic Type	Coordinates (Angstroms) X	Y	Z
1	6	0	3.814351	1.089866	-0.076089
2	6	0	2.431327	0.956560	-0.098753
3	6	0	1.847389	-0.314857	-0.036457
4	6	0	2.674550	-1.440699	0.041922

5	6	0	4.060422	-1.299345	0.064152
6	6	0	4.646149	-0.033828	0.006101
7	6	0	-2.540278	-1.113672	-0.217654
8	6	0	-3.896446	-1.365797	-0.195432
9	6	0	-4.754860	-0.284170	-0.016025
10	6	0	-4.281475	1.009628	0.123204
11	6	0	-2.907325	1.263429	0.099232
12	6	0	-2.001132	0.175400	-0.059042
13	6	0	0.400493	-0.534050	-0.049942
14	6	0	-0.560384	0.408753	-0.048899
15	7	0	-2.468495	2.564937	0.303433
16	7	0	-6.195428	-0.513099	0.012412
17	8	0	-6.939808	0.454537	0.174505
18	8	0	-6.604505	-1.666267	-0.126611
19	8	0	4.286930	2.367592	-0.141883
20	6	0	5.695447	2.583044	-0.112090
21	8	0	4.772331	-2.458927	0.143589
22	6	0	6.195356	-2.388196	0.181627
23	1	0	1.835672	1.856877	-0.170253
24	1	0	2.250680	-2.436369	0.089954
25	1	0	5.718063	0.077532	0.020901
26	1	0	-1.870020	-1.949770	-0.366790
27	1	0	-4.288687	-2.364333	-0.315175
28	1	0	-4.982380	1.823509	0.249819
29	1	0	0.113982	-1.581007	-0.046952
30	1	0	-0.239374	1.442537	-0.014328
31	1	0	-1.603001	2.839116	-0.139133
32	1	0	-3.178884	3.276728	0.188043
33	1	0	5.830644	3.660906	-0.167395
34	1	0	6.134535	2.210718	0.817659
35	1	0	6.188000	2.112660	-0.967605
36	1	0	6.541107	-3.417194	0.249850
37	1	0	6.541119	-1.830956	1.056752
38	1	0	6.594904	-1.931689	-0.728193

Visualization of calculated geometry of (**6b**):

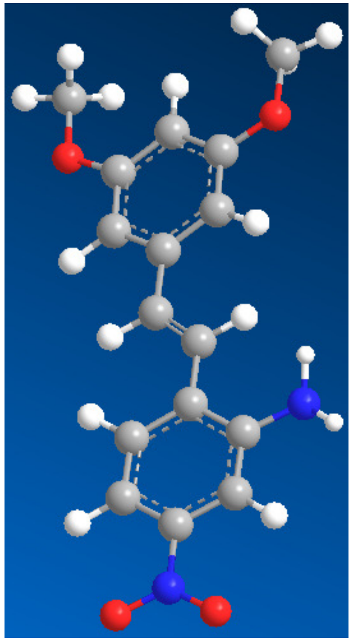

The calculated coordinates of (**7b**)(the part of calculated log file):

Standard orientation:

Center Number	Atomic Number	Atomic Type	Coordinates (Angstroms) X	Y	Z
1	6	0	-3.312225	1.081979	0.094523
2	6	0	-1.932889	0.958396	0.116671
3	6	0	-1.321270	-0.295073	-0.035339
4	6	0	-2.144128	-1.418614	-0.189983
5	6	0	-3.527822	-1.303511	-0.214151
6	6	0	-4.130174	-0.046735	-0.084236
7	6	0	3.045161	-1.060320	0.338571
8	6	0	4.398789	-1.328643	0.337905
9	6	0	5.266333	-0.282952	0.036140
10	6	0	4.806248	0.992017	-0.248854
11	6	0	3.434920	1.260631	-0.250918
12	6	0	2.519822	0.208704	0.038817
13	6	0	0.125567	-0.495457	-0.039167
14	6	0	1.080445	0.450841	0.025004
15	7	0	3.008110	2.528154	-0.618283
16	8	0	-4.286306	-2.425788	-0.443378
17	6	0	-5.035161	-2.899493	0.687933
18	8	0	-5.494407	0.079047	-0.102516
19	6	0	-6.047706	0.336109	-1.405550
20	8	0	-3.876232	2.332743	0.176185
21	6	0	-4.494614	2.636825	1.436985
22	7	0	6.704567	-0.528882	0.032479
23	8	0	7.458431	0.405631	-0.240875
24	8	0	7.101919	-1.662985	0.300779
25	1	0	-1.351696	1.861629	0.251473
26	1	0	-1.710169	-2.405018	-0.305561
27	1	0	2.365328	-1.860521	0.598228
28	1	0	4.781822	-2.309722	0.573032
29	1	0	5.514363	1.777279	-0.474054
30	1	0	0.425997	-1.535264	-0.121446
31	1	0	0.759194	1.485646	0.057495
32	1	0	2.125780	2.852061	-0.249209
33	1	0	3.715735	3.250525	-0.569927
34	1	0	-5.586064	-3.772450	0.342644
35	1	0	-5.732054	-2.138314	1.041220

36	1	0	-4.357471	-3.191079	1.495446
37	1	0	-7.124484	0.416822	-1.268473
38	1	0	-5.292091	1.927039	1.662845
39	1	0	-5.823417	-0.488930	-2.085496
40	1	0	-4.910146	3.637998	1.337948
41	1	0	-5.655944	1.272229	-1.809613
42	1	0	-3.748421	2.627746	2.236395

Visualization of calculated geometry of (**7b**):

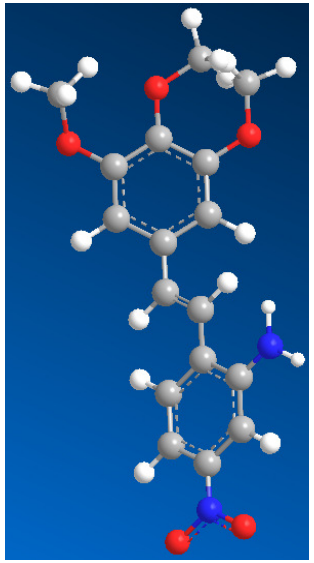

References in supplementary materials:

[1] H. Krawczyk, K. Janowska, R. Matczak, J. Zakrzewski.*Tet. Lett*. **2012**, *53,* 6504-6507.

[2] M.J. Frisch, G.W. Trucks, H.B. Schlegel, G.E. Scuseria, M.A. Robb, J.R. Cheeseman,J.A. Montgomery, T. Vreven, K.N. Kudin, J.C. Burant, J.M. Millam, S.S. Iyengar, J.Tomasi, V. Barone, B. Mennucci, M. Cossi, G. Scalmani, N. Rega, G.A. Petersson, H. Nakatsuji, M. Hada, M. Ehara, K. Toyota, R. Fukuda, J. Hasegawa, M. Ishida, T.Nakajima, Y. Honda, O. Kitao, H. Nakai, M. Klene, X. Li, J.E. Knox, H.P. Hratchian, J.B. Cross, V. Bakken, C. Adamo, J. Jaramillo, R. Gomperts, R.E. Stratmann, O. Yazyev, A.J. Austin, R. Cammi, C. Pomelli, J.W. Ochterski, P.Y. Ayala, K. Morokuma, G.A. Voth, P. Salvador, J.J. Dannenberg, V.G. Zakrzewski, S. Dapprich, A.D. Daniels, M.C. Strain, O. Farkas, D.K. Malick, A.D. Rabuck, K.Raghavachari, J.B. Foresman, J.V. Ortiz, Q. Cui, A.G. Baboul, S. Clifford, J. Cioslowski, B.B. Stefanov, G. Liu, A. Liashenko, P. Piskorz, I. Komaromi, R.L. Martin, D.J. Fox, T. Keith, M.A. Al-Laham, C.Y. Peng, A. Nanayakkara, M. Challacombe, P.M.W. Gill, B. Johnson, W. Chen, M.W. Wong, C. Gonzalez, J.A. Pople, Gaussian Inc., Wallingford, CT, **2004** (Gaussian 03, Revision E.01).

[3] J. Tomasi, B. Mennucci, R. Cammi*, Chem. Rev*. **2005**, *105*, 2999–3093.

Proton spectrum of (**1b**)

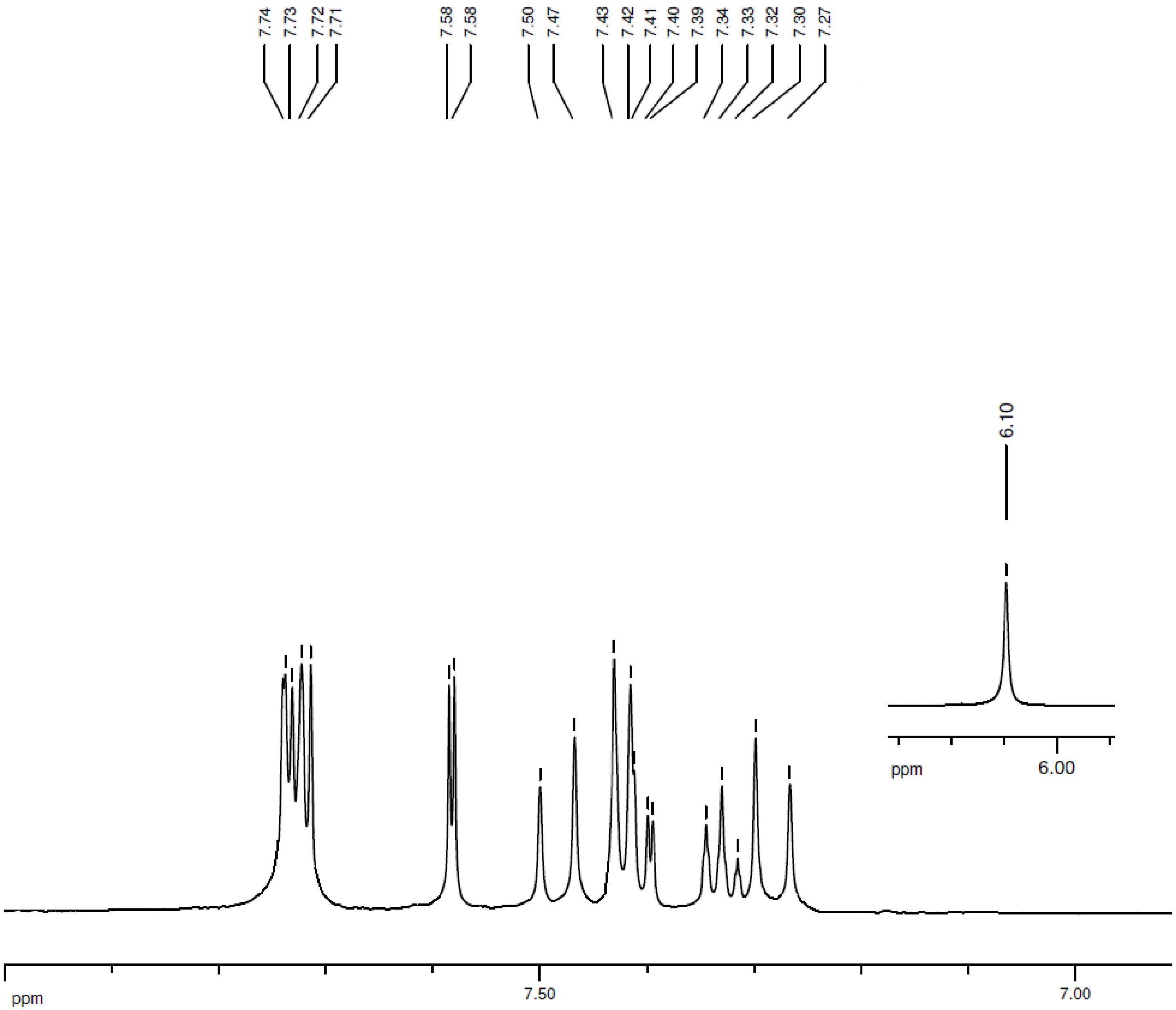

^{13}C spectrum of (**1b**)

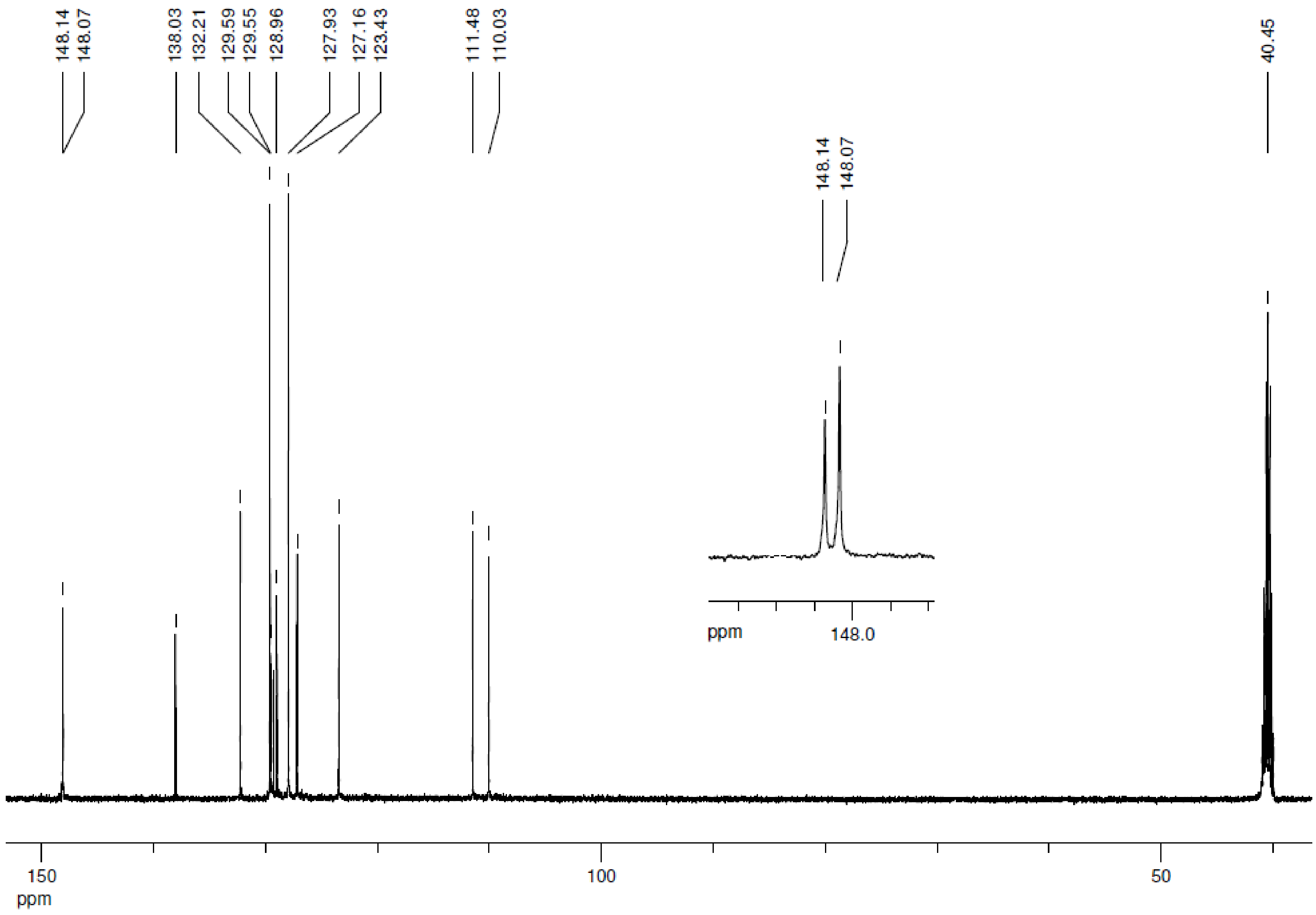

Proton spectrum of (**2b**)

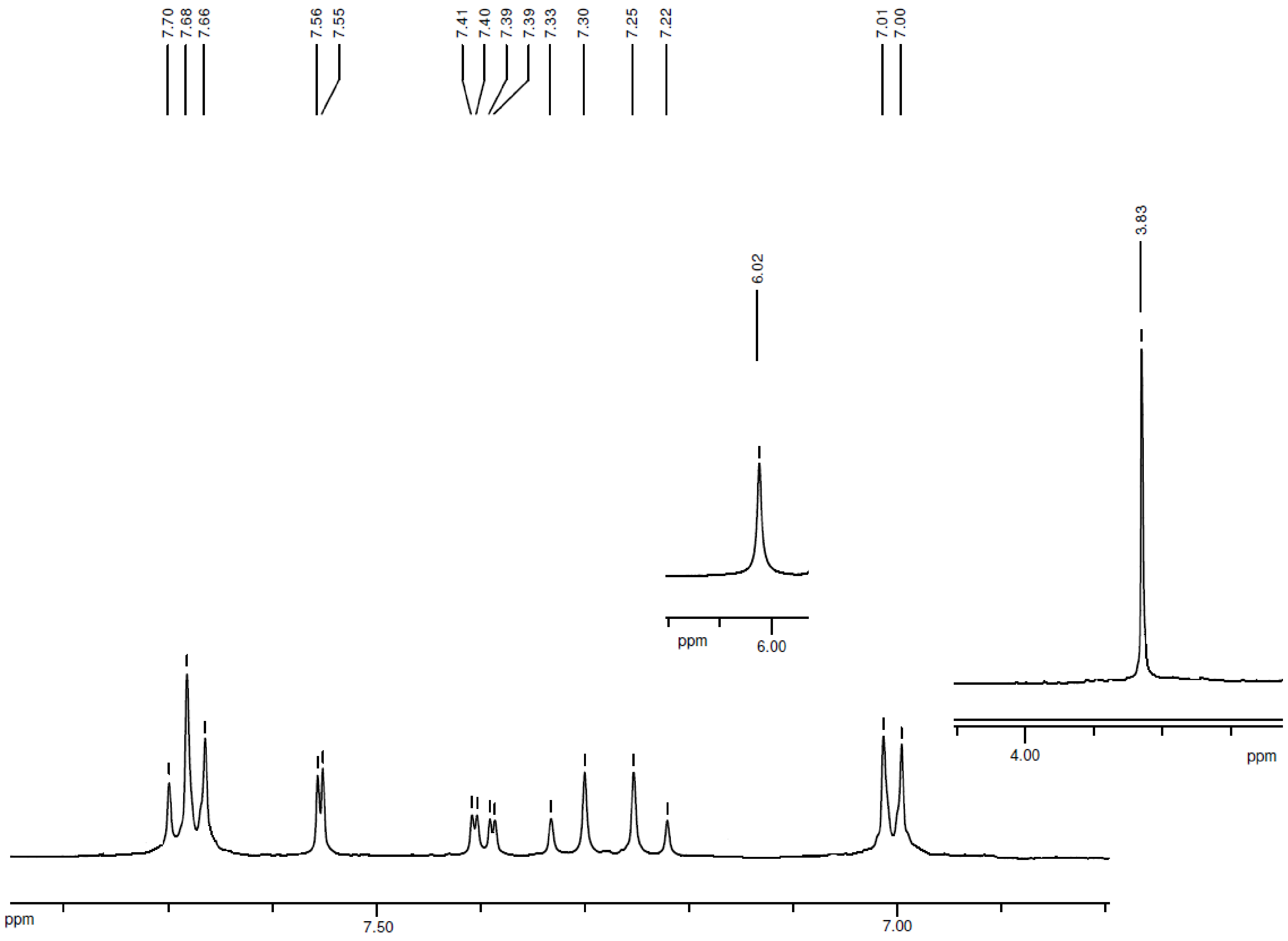

^{13}C spectrum of (**2b**)

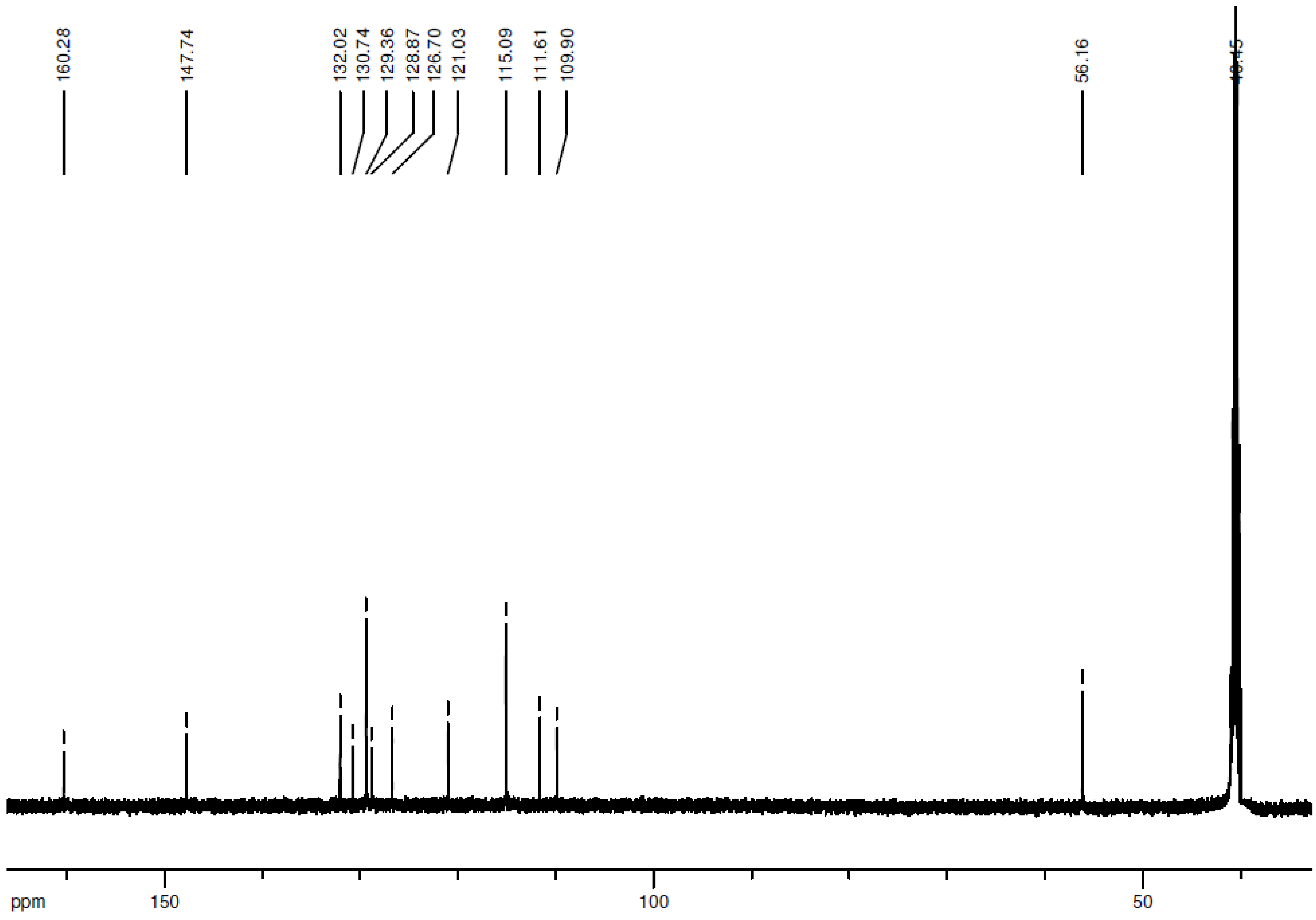

Proton spectrum of (**3b**)

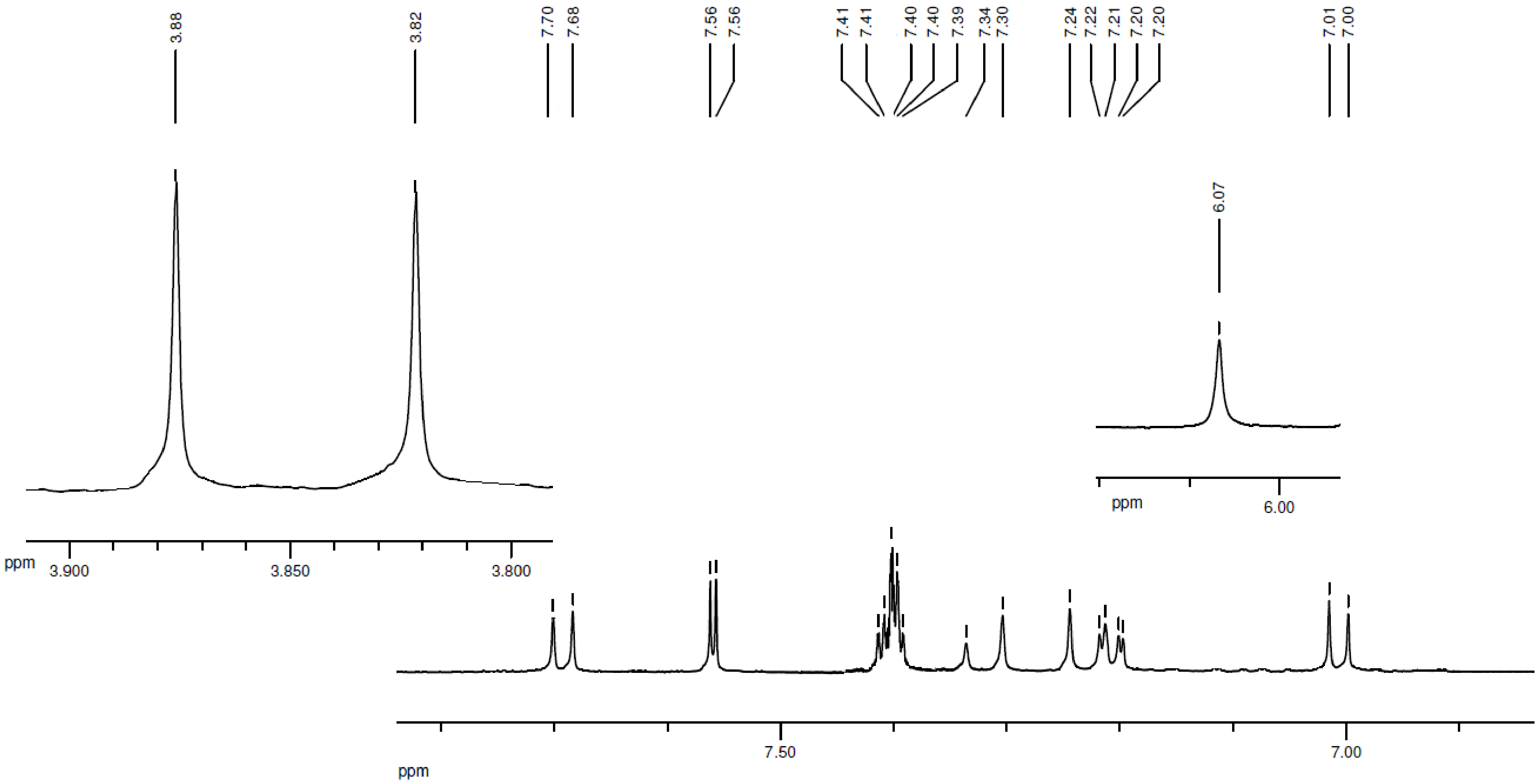

^{13}C spectrum of (**3b**)

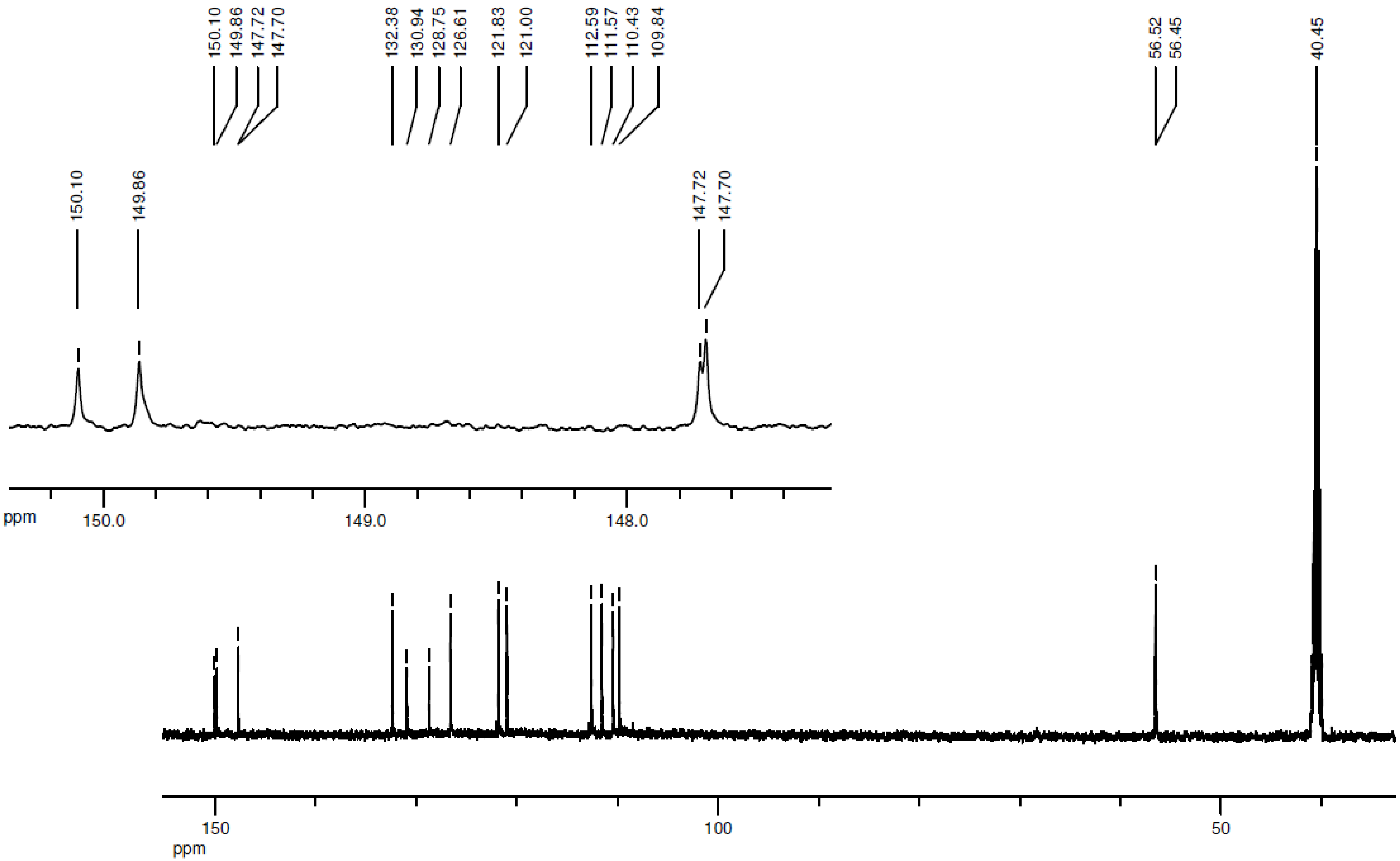

Proton spectrum of (**4b**)

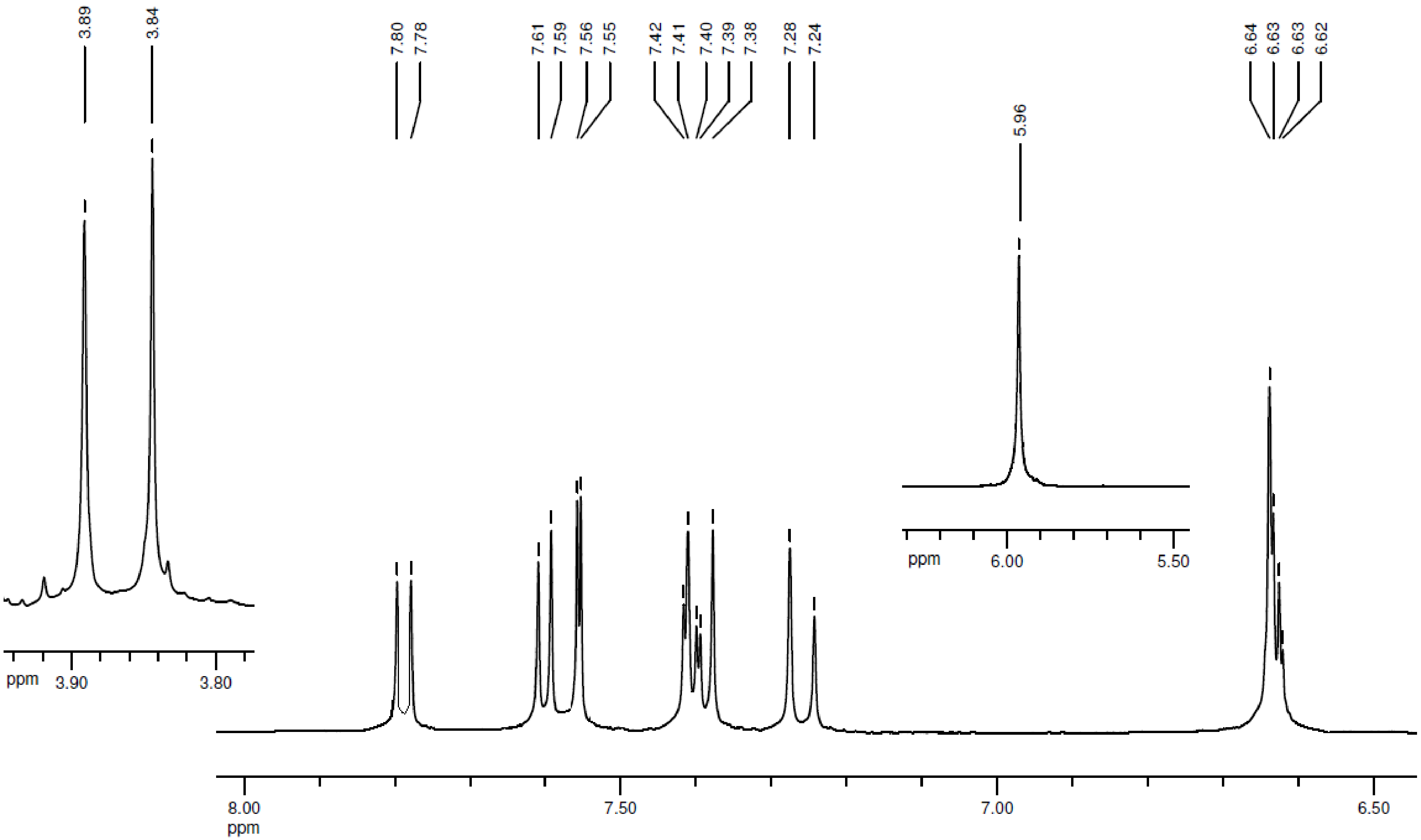

^{13}C spectrum of (**4b**)

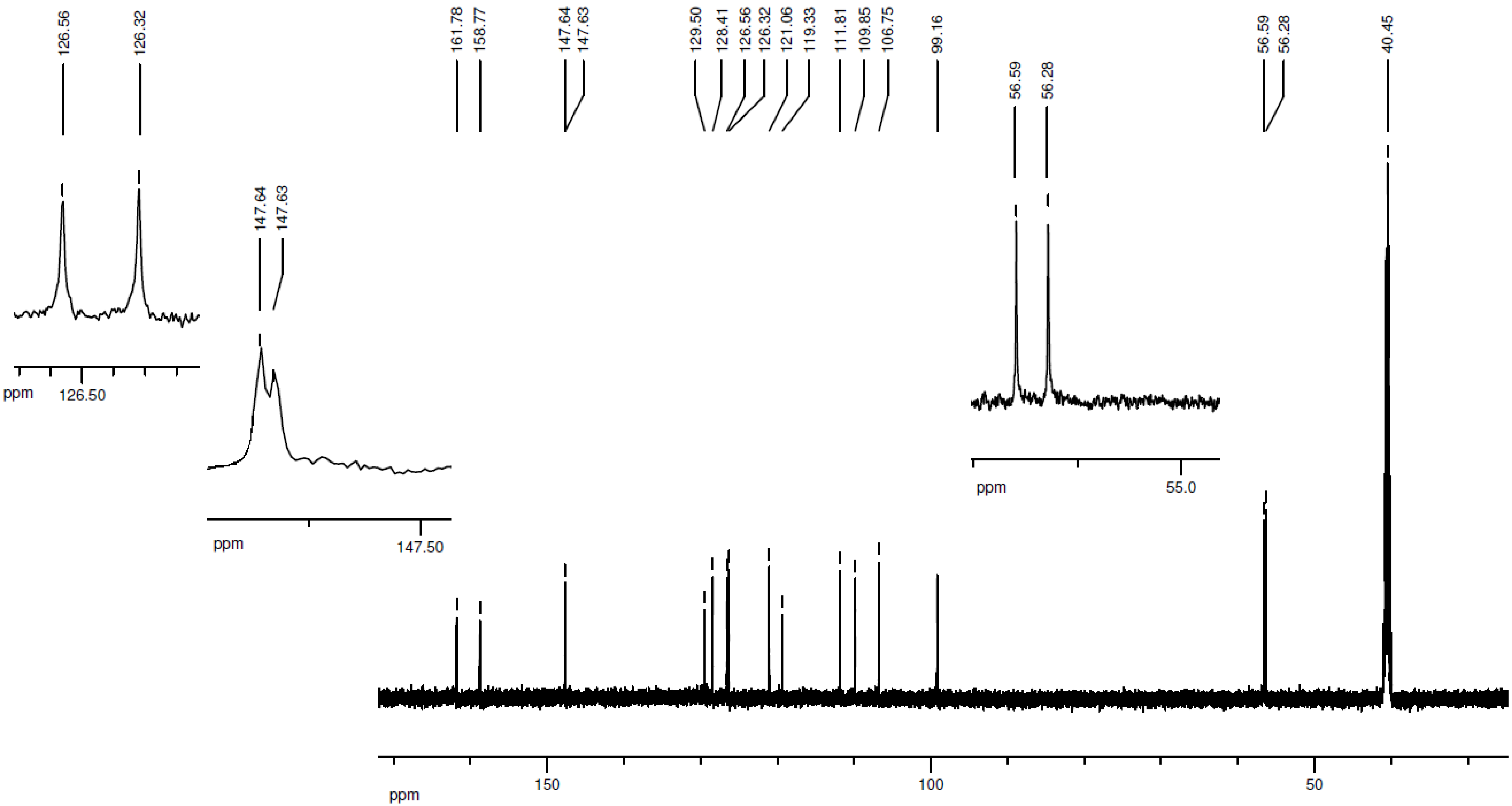

Proton spectrum of (**5b**)

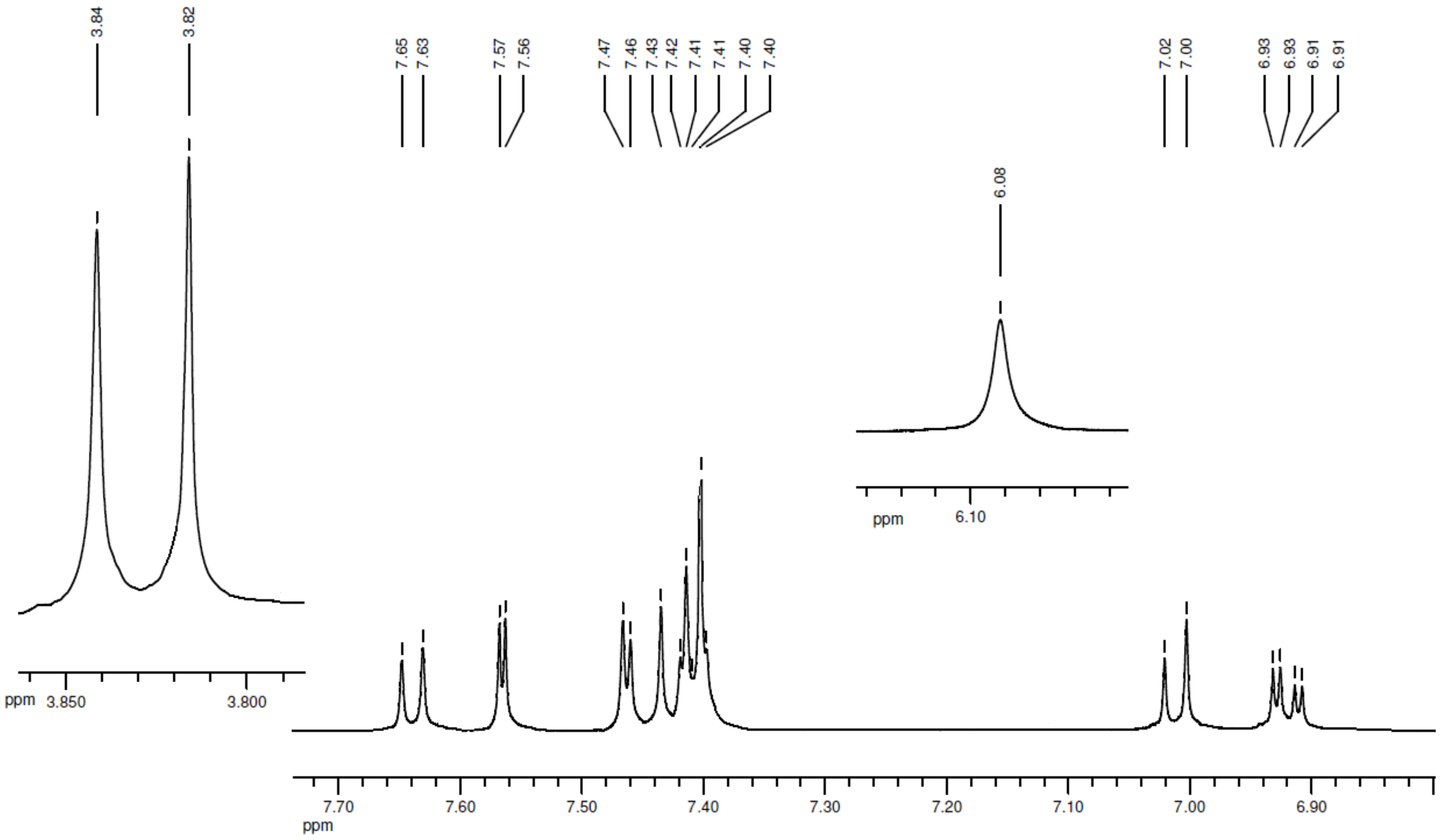

^{13}C spectrum of (**5b**)

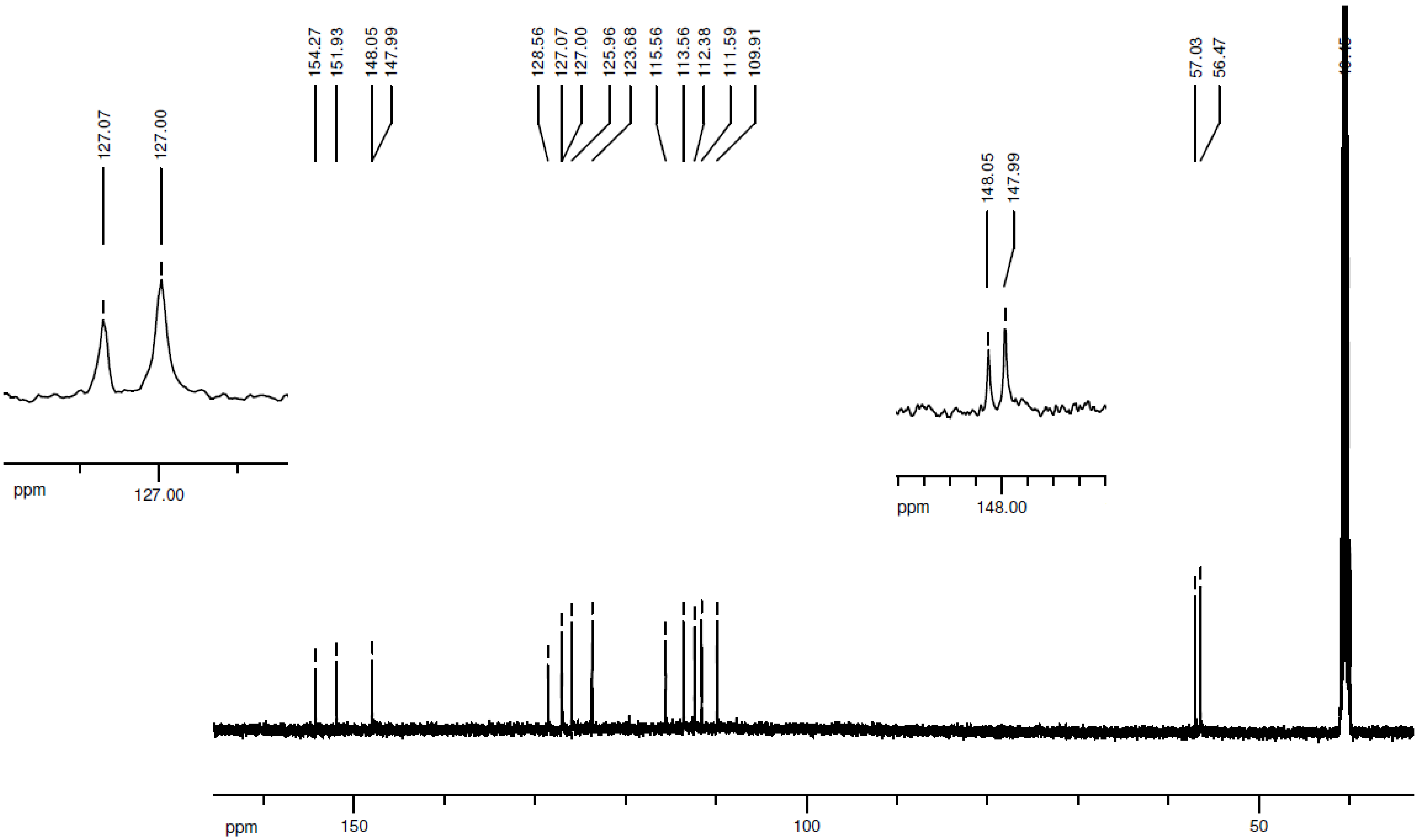

Proton spectrum of (**6b**)

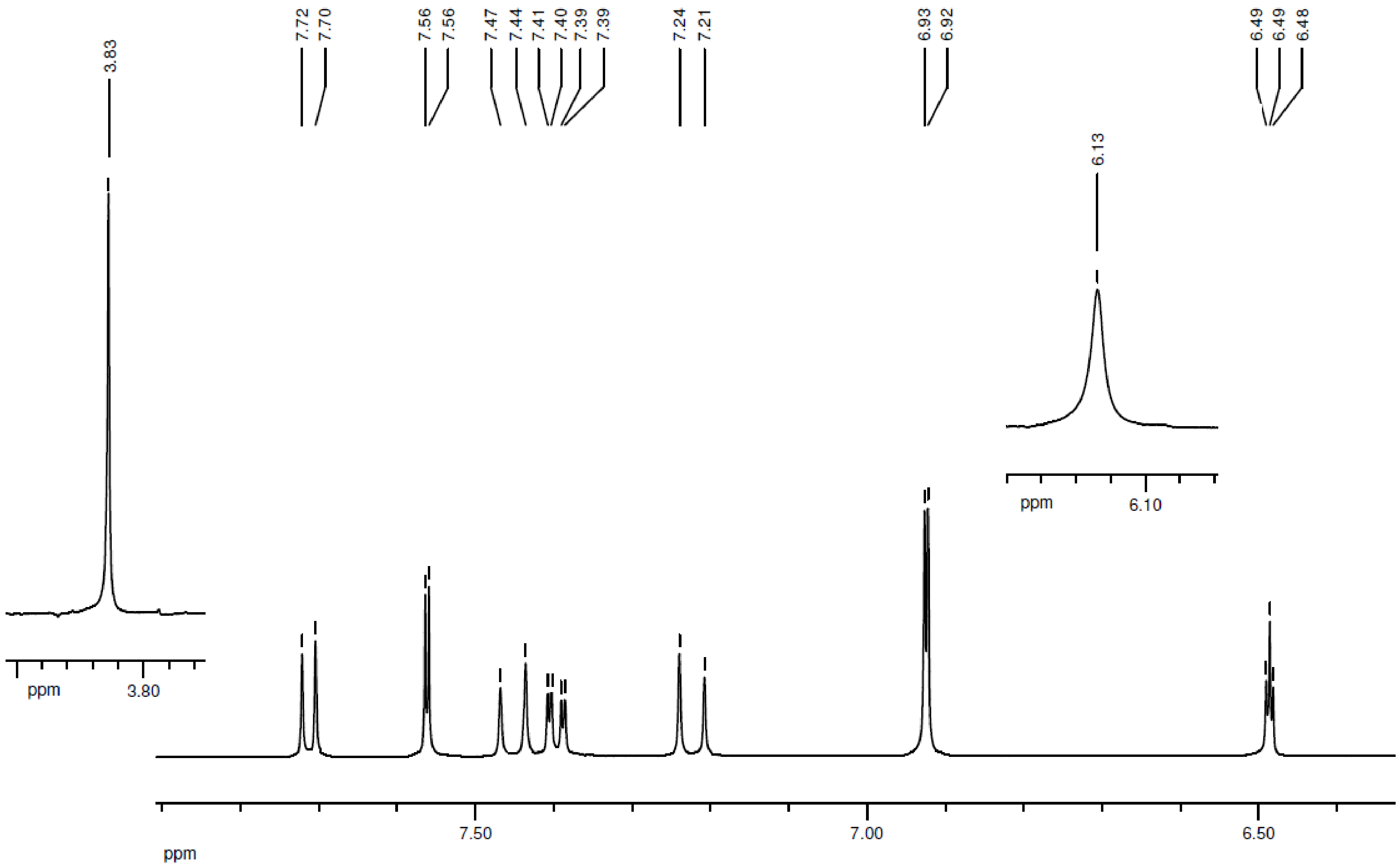

^{13}C spectrum of (**6b**)

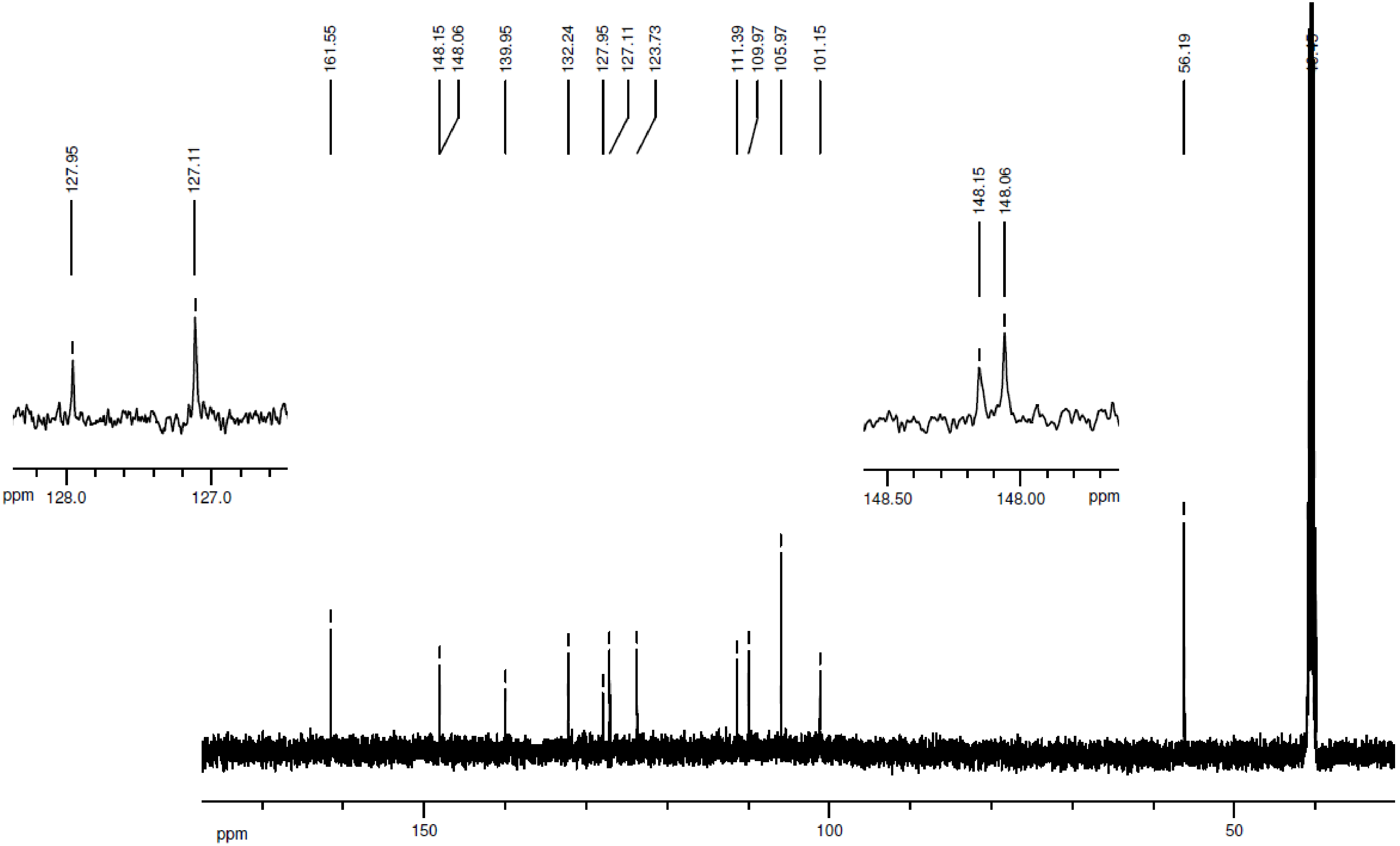

Proton spectrum of (**7b**)

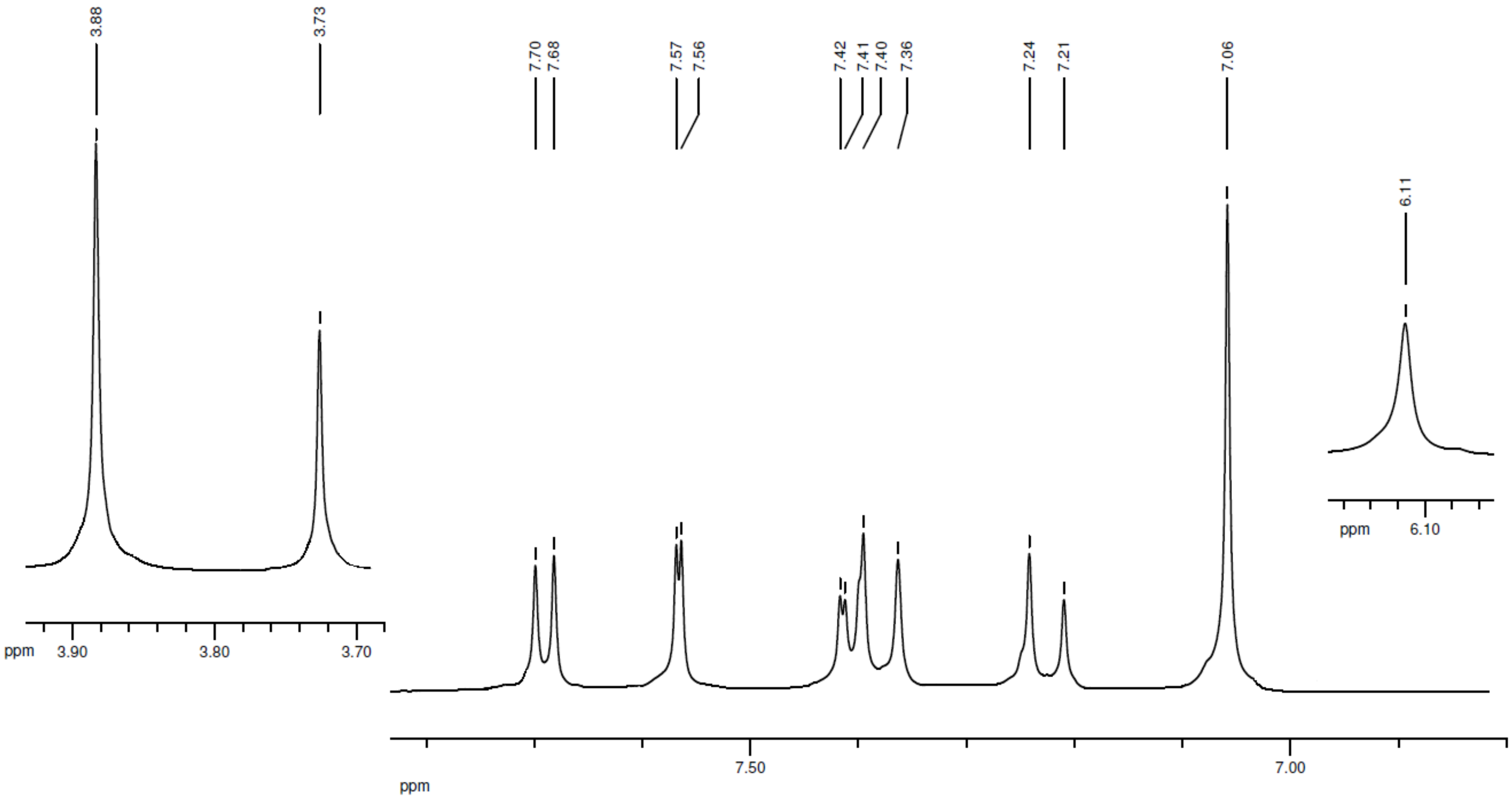

^{13}C spectrum of (**7b**)

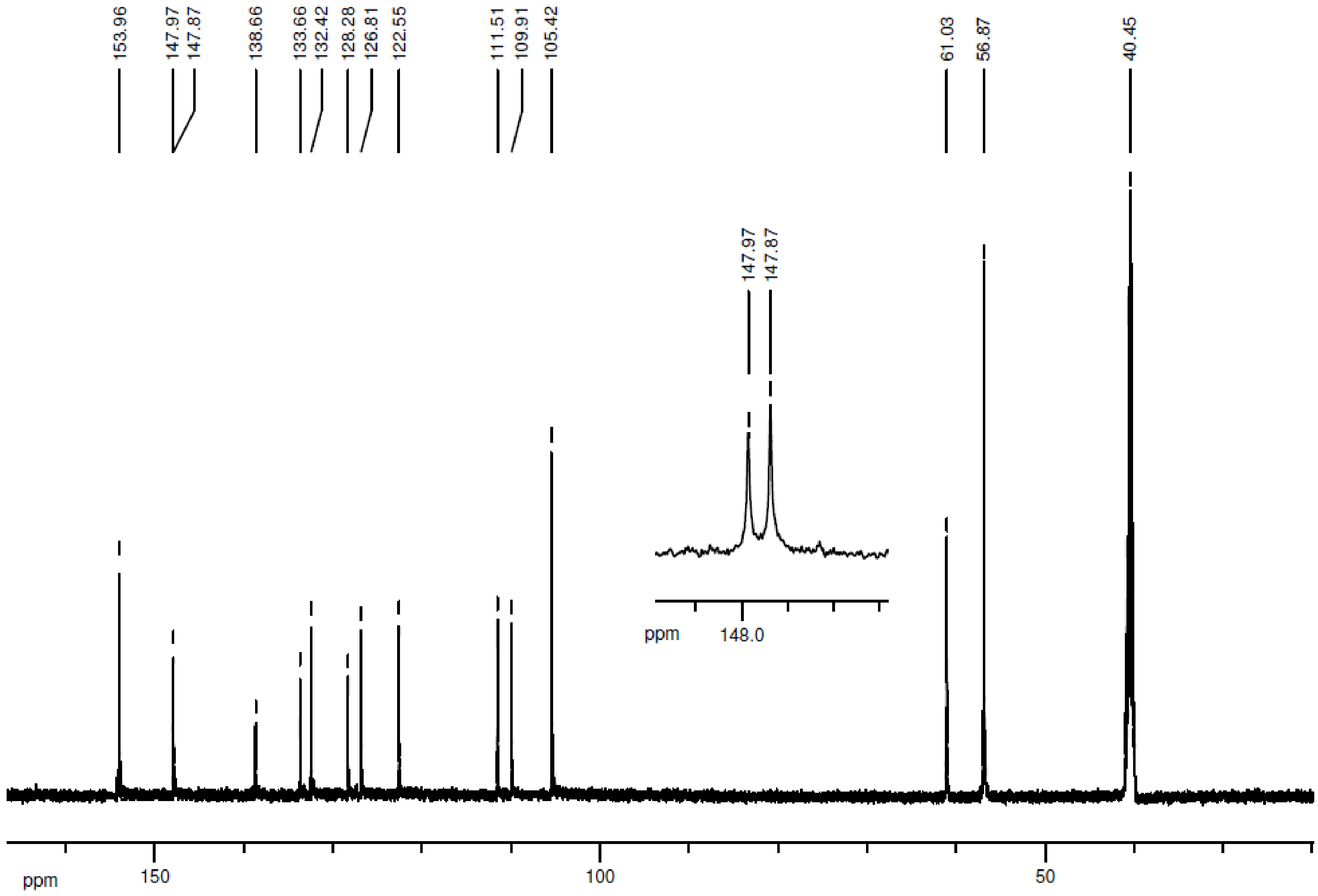

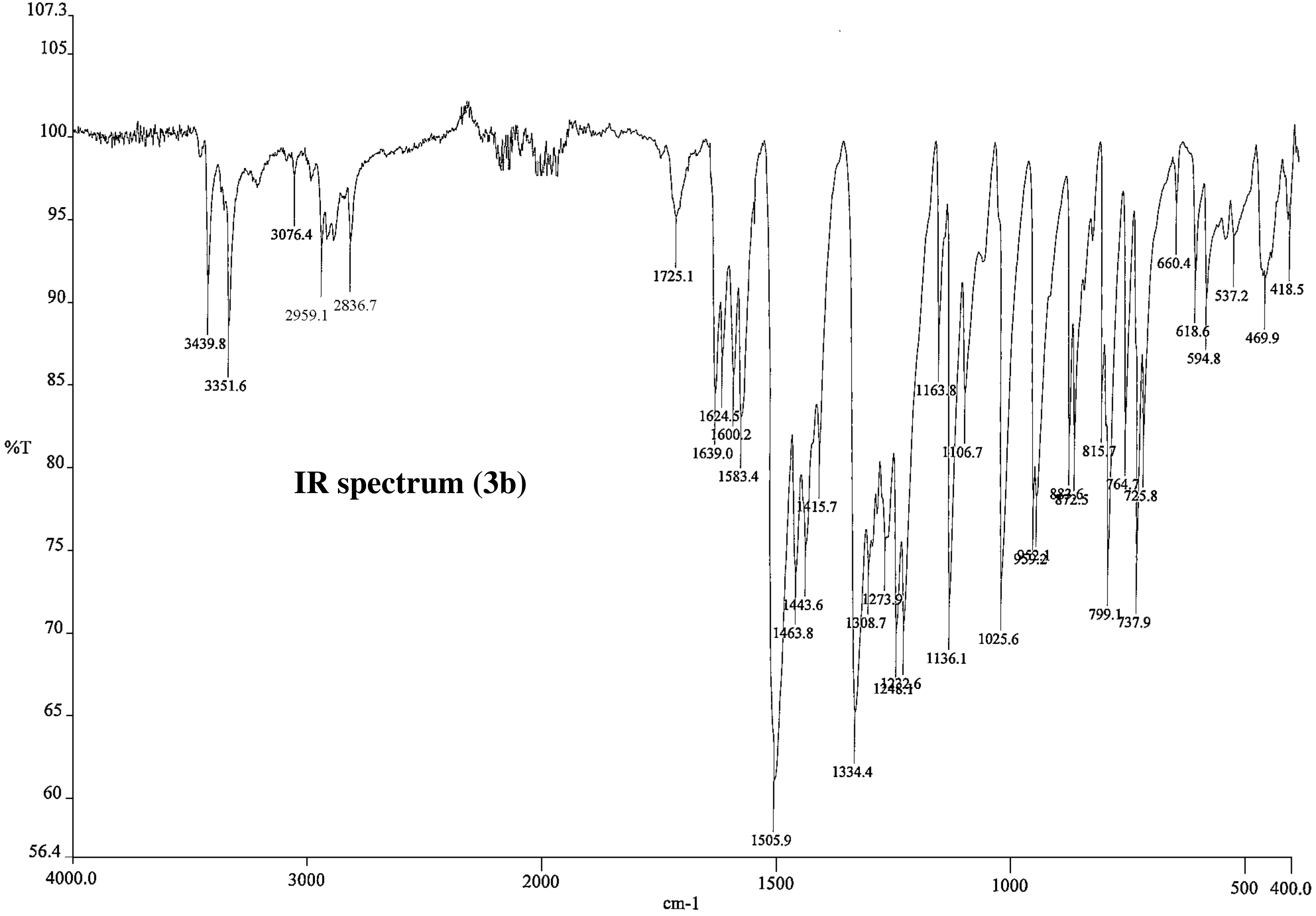
IR spectrum (3b)
%T
cm-1
107.3
105
100
95
90
85
80
75
70
65
60
56.4
4000.0
3000
2000
1500
1000
500
400.0
3439.8
3351.6
3076.4
2959.1
2836.7
1725.1
1639.0
1624.5
1600.2
1583.4
1505.9
1463.8
1443.6
1415.7
1334.4
1308.7
1273.9
1248.1
1232.6
1163.8
1136.1
1106.7
1025.6
959.2
952.1
882.6
872.5
815.7
799.1
764.7
737.9
725.8
660.4
618.6
594.8
537.2
469.9
418.5

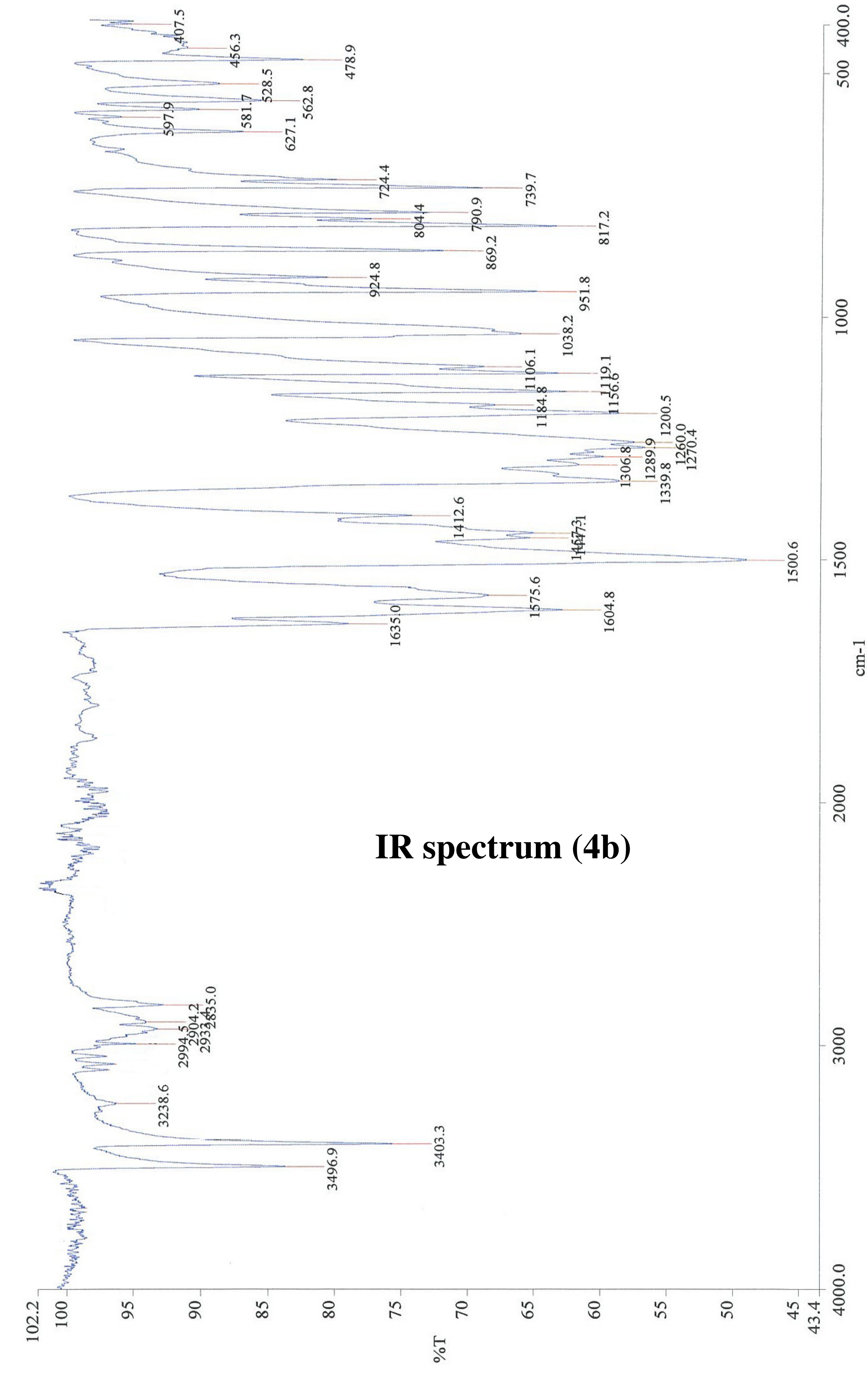

IR spectrum (4b)

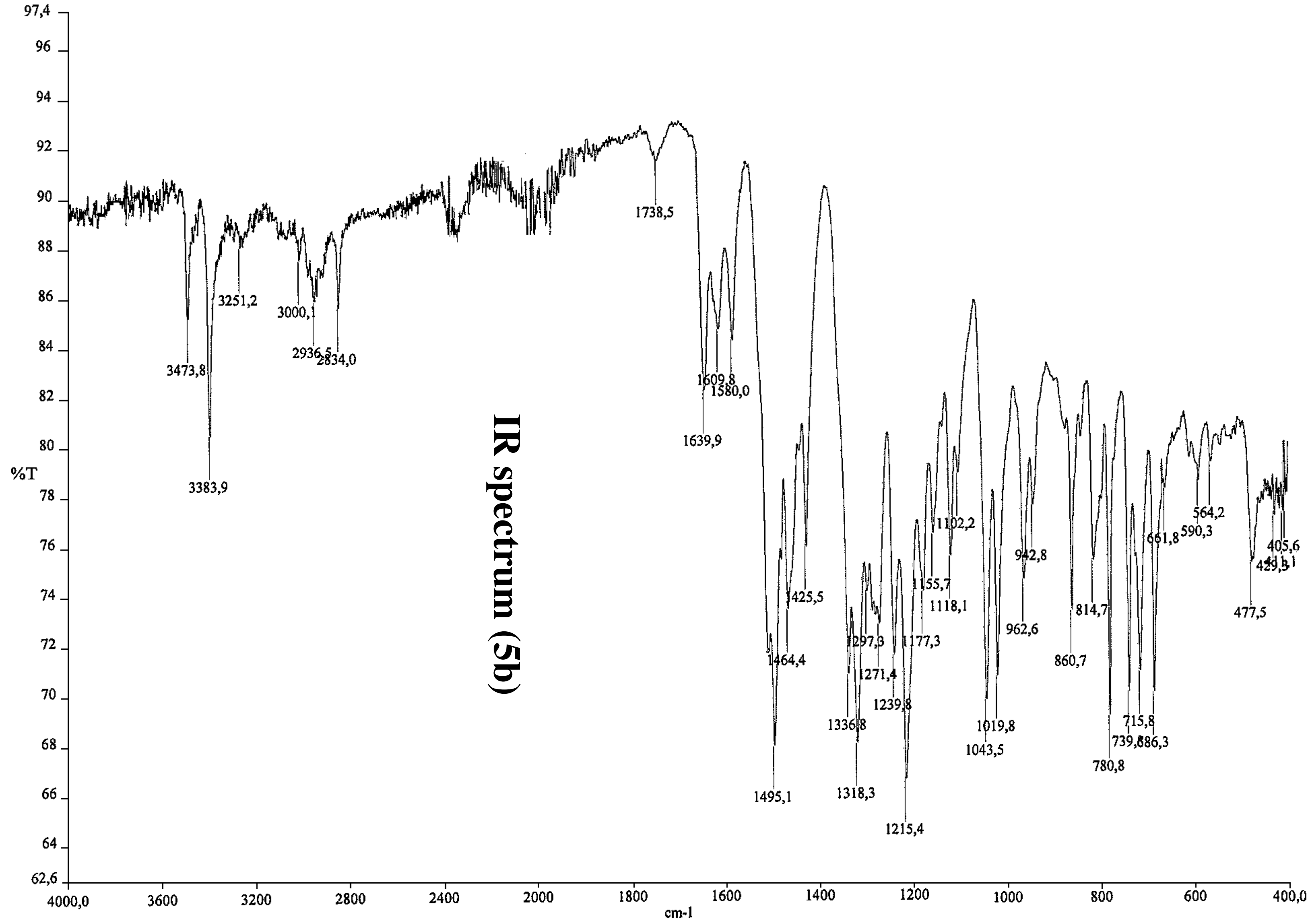

IR spectrum (5b)

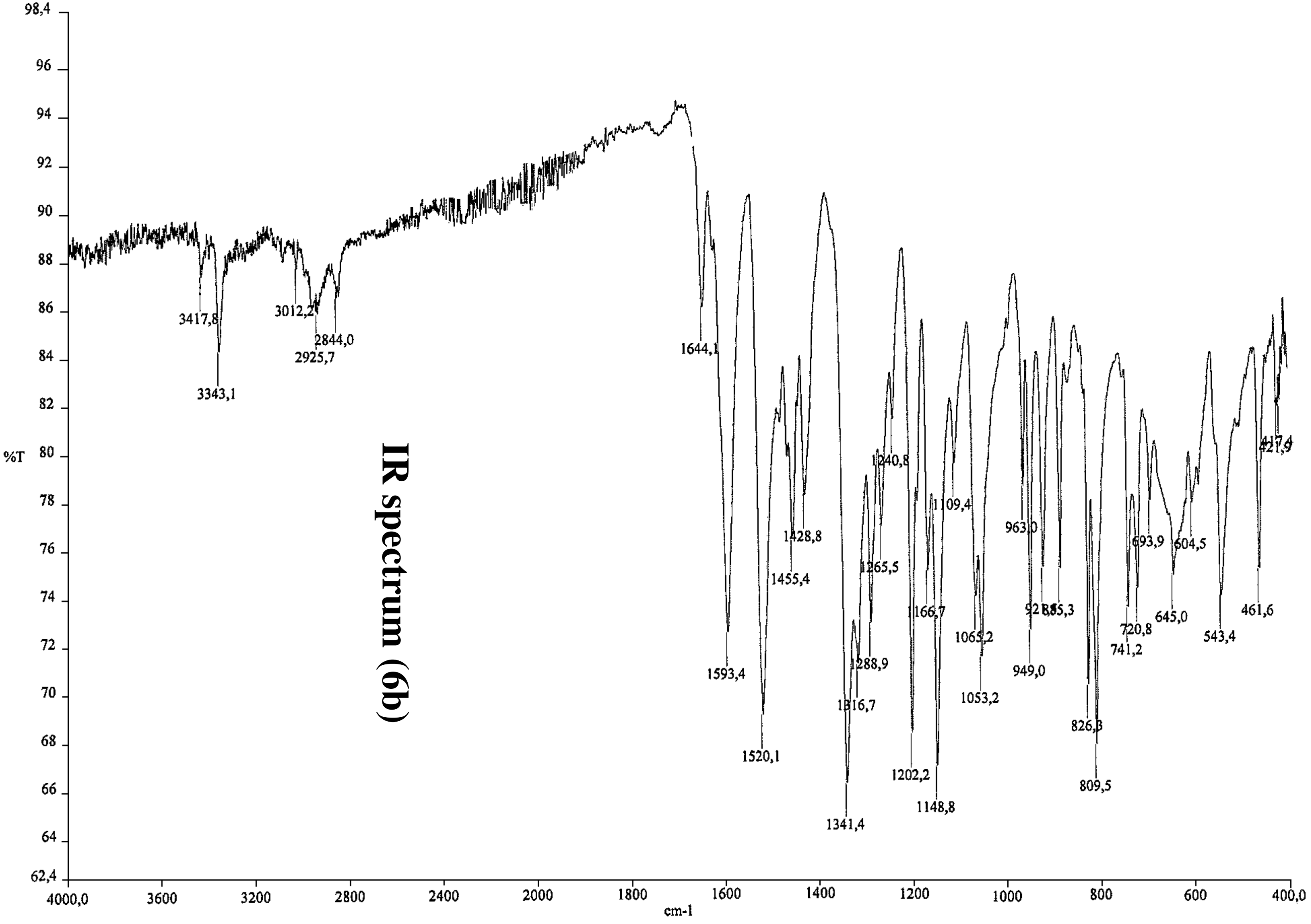

IR spectrum (6b)

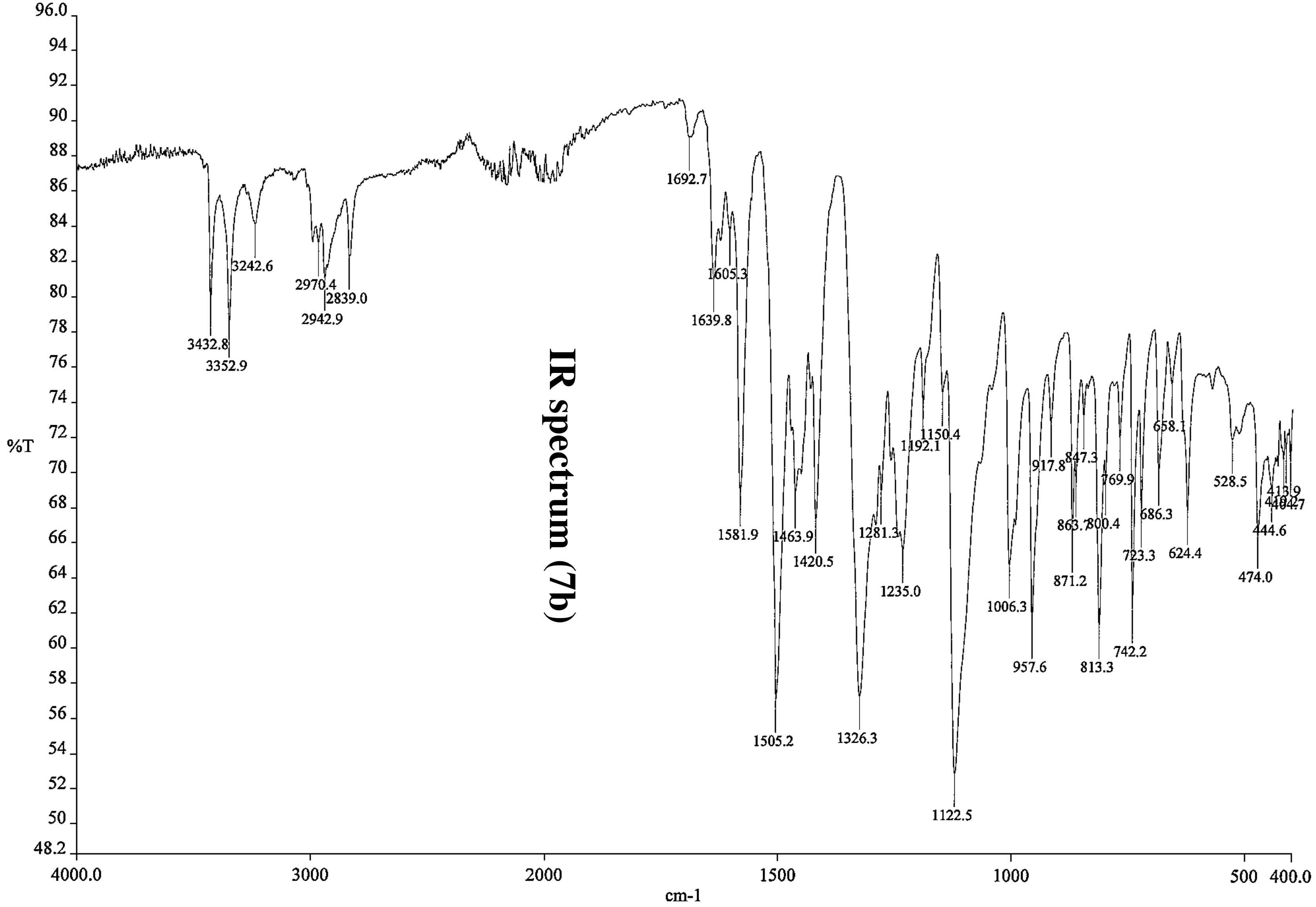
IR spectrum (7b)
%T
cm-1
96.0
94
92
90
88
86
84
82
80
78
76
74
72
70
68
66
64
62
60
58
56
54
52
50
48.2
4000.0
3000
2000
1500
1000
500
400.0
3432.8
3352.9
3242.6
2970.4
2942.9
2839.0
1692.7
1639.8
1605.3
1581.9
1505.2
1463.9
1420.5
1326.3
1281.3
1235.0
1192.1
1150.4
1122.5
1006.3
957.6
917.8
871.2
863.7
847.3
813.3
800.4
769.9
742.2
723.3
686.3
658.1
624.4
528.5
474.0
444.6
413.9
